# Adaptations of archaeal and bacterial membranes to variations in temperature, pH and pressure

**DOI:** 10.1007/s00792-017-0939-x

**Published:** 2017-05-15

**Authors:** Melvin F. Siliakus, John van der Oost, Servé W. M. Kengen

**Affiliations:** Laboratory of Microbiology, Wageningen University and Research, Stippeneng 4, 6708 WE Wageningen, The Netherlands

**Keywords:** Archaea, Bacteria, Membranes, Adaptation, Lipids

## Abstract

The cytoplasmic membrane of a prokaryotic cell consists of a lipid bilayer or a monolayer that shields the cellular content from the environment. In addition, the membrane contains proteins that are responsible for transport of proteins and metabolites as well as for signalling and energy transduction. Maintenance of the functionality of the membrane during changing environmental conditions relies on the cell’s potential to rapidly adjust the lipid composition of its membrane. Despite the fundamental chemical differences between bacterial ester lipids and archaeal ether lipids, both types are functional under a wide range of environmental conditions. We here provide an overview of archaeal and bacterial strategies of changing the lipid compositions of their membranes. Some molecular adjustments are unique for archaea or bacteria, whereas others are shared between the two domains. Strikingly, shared adjustments were predominantly observed near the growth boundaries of bacteria. Here, we demonstrate that the presence of membrane spanning ether-lipids and methyl branches shows a striking relationship with the growth boundaries of archaea and bacteria.

**Electronic supplementary material:**

The online version of this article (doi:10.1007/s00792-017-0939-x) contains supplementary material, which is available to authorized users.

## Introduction

**Fig. 1:**
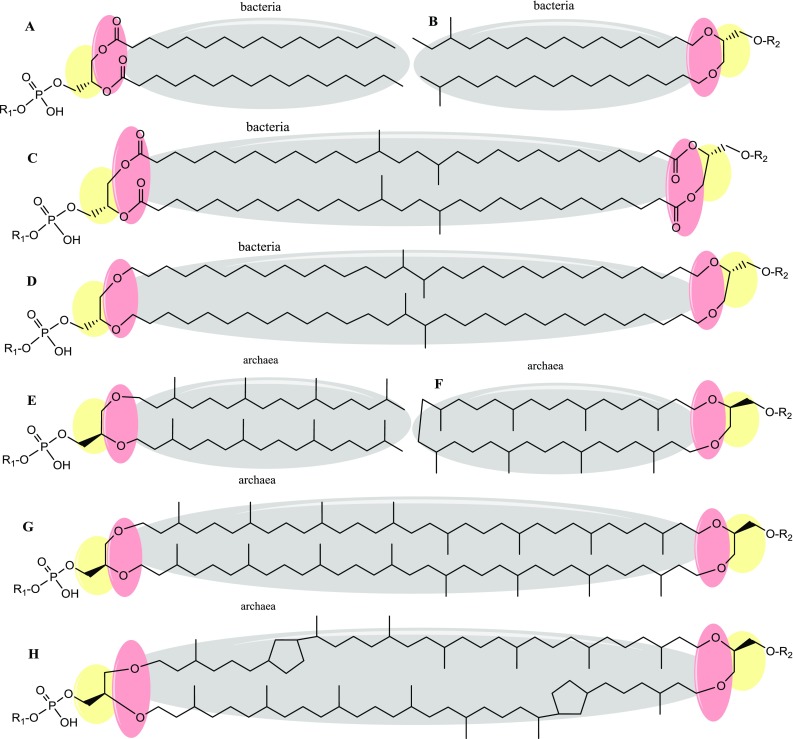
Common bacterial and archaeal lipid variations and the lipid divide. The lipid divide is presented by three colours. *Grey* hydrocarbon chains are represented by fatty acids in bacteria and isoprenoid chains in archaea. *Red* in bacteria, ester-bonds typically link the hydrocarbon chains to the glycerol backbone. In archaea, hydrocarbon chains are attached to the glycerol backbone by ether-bonds. *Yellow* the backbone moiety in bacterial lipids is represented by glycerol-3-phosphate (**a**–**d**). In archaeal lipids the backbone moiety is represented by the enantiomeric glycerol-1-phosphate (**e**–**h**). Common variations on the bacterial ‘phosphatidic acid’ (**a**) are presented by anteiso- and iso-branched chain fatty acids or ether bonds shown in (**b**). **c** and **d** show branched chain GDGTs with iso-diabolic acid and diabolic acid with either ester or ether bonds, respectively. Common variations on the archaeal ‘archaetidic acid’ (**e**) are presented by a fusion of the isoprenoid tail ends to form macrocyclic archaeol (**f**). Archaeal bipolar glycerol dialkyl glycerol tetraether (GDGT-0) is depicted in **g** and spans the membrane to form lipid monolayers. **h** shows GDGT-2, this bipolar lipid contains 2 cyclopentane rings in the phytanyl chains. Head groups are presented either by *R1* phosphate polar heads, or *R2* single or multiple hexoses

The core lipids that serve as the framework for fully mature membrane lipids are fundamentally different in bacteria and archaea. These differences are the basis of the so-called ‘lipid divide’ and are represented by ‘phosphatidic acid’ for bacteria and eukarya and ‘archaetidic acid’ for archaea. Phosphatidic acid is composed of two fatty acid hydrocarbon chains esterified to the sn-1 and sn-2 position of glycerol-3-phosphate (G-3-P) (Fig. 1a). Archaetidic acid (also known as di-*O*-geranylgeranylglyceryl phosphate; DGGGP) on the other hand consists of two methyl-branched isoprenoids (phytanyls once saturated) connected by ether-bonds to the *sn*-2 and *sn*-3 position of glycerol-1-phosphate (G-1-P) (Fig. 1e). Besides the typical diester and diether lipids, most archaea but also some bacteria also contain glycerol-dialkyl-glycerol-tetraether (GDGT) lipids in their membranes (Fig. 1c, d, g, h). These lipids are bipolar and believed to be the product of a tail-to-tail condensation between two lipids and as such form a monolayer instead of a bilayer membrane. Although the model core lipids have been dubbed to be strictly domain-specific, a certain degree of overlap exists between these traits. We now know that bacteria occasionally also produce membrane spanning ether lipids (Weijers et al. 2006) and archaea also produce fatty acid ether lipids (Gattinger et al. 2002). Because of the general chemical differences, bacteria and archaea also evolved domain-specific adaptation mechanisms to effectively respond to different physicochemical conditions of their habitats. The common physicochemical parameters that breach the integrity of membranes are temperature, pH and hydrostatic pressure. Typical membrane characteristics that are adversely affected by environmental changes are the permeability and fluidity. These parameters have, for example, a large effect on the function and mobility of membrane proteins, diffusion of nutrients and proper separation during cell division. To maintain physiological homeostasis, membrane integrity is, therefore, continuously secured through a mechanism called ‘homeoviscous adaptation’. This process was first demonstrated in *Escherichia coli* by the observation that fluidity of the membrane remains relatively constant at various temperatures (Sinensky 1974). The cells manage this by actively modifying their lipid composition to maintain membrane functionality at different temperatures. These modifications often cause shifts in ratios of lipid types and/or their hydrocarbon moieties, rather than complete replacement of certain species. Additionally, it has been demonstrated that polar head groups also play a significant role in the maintenance of membrane fluidity and permeability. Both for bacteria and archaea a change in polar head group composition has been observed in response to changing environmental conditions. Despite this pivotal role, changes in polar head groups have not been documented properly since the discovery of this mechanism. Although the core lipids were once thought to form a sharp distinction between the two domains, recent analyses have revealed that a certain degree of overlap exists between some lipids features which in some cases can be regarded as a form of homeoviscous adaptation.

We here provide an overview of adaptations of the core-lipids in bacterial and archaeal cells in response to changes in physicochemical conditions. Additionally, we present a comparative analysis of the reported growth ranges of the most robust extremophiles to date, to disclose the growth boundaries of the bacterial and archaeal domains. We further discuss these boundaries in relation to the encountered lipid compositions and their adaptation in the archaea and bacteria. Adaptation of cell physiology to physicochemical conditions occurs at two levels, long-term (genome evolution, defines the range within which a cell can survive) and short-term (reversible regulation of gene expression and enzyme activity to achieve optimal functionality). A membrane adaptation can thus refer to the generally encountered lipid composition that enables a species to thrive at a particular challenging habitat within its optimum. This kind of adaptation is regarded as a native phenotype that contributes to the robustness against a particular challenge/parameter and is therefore, termed ‘physiological membrane adaptation’. Alternatively, an adaptation can also involve the changes in the lipid composition when conditions of the natural habitats change. This kind of adaptation is regarded as a stress response to the physicochemical change beyond the organism’s optimum that aids in the survival of the cell. This change will, therefore, be termed here as ‘membrane stress response’ and generally resembles the permanent physiological membrane adaptations to some extent. While most membrane adaptation studies have focussed on the changes that have been observed in the core lipid, it has become more apparent that major changes are also elicited on the polar head groups as adaptive traits.

### Low temperature adaptation in bacteria

**Fig. 2:**
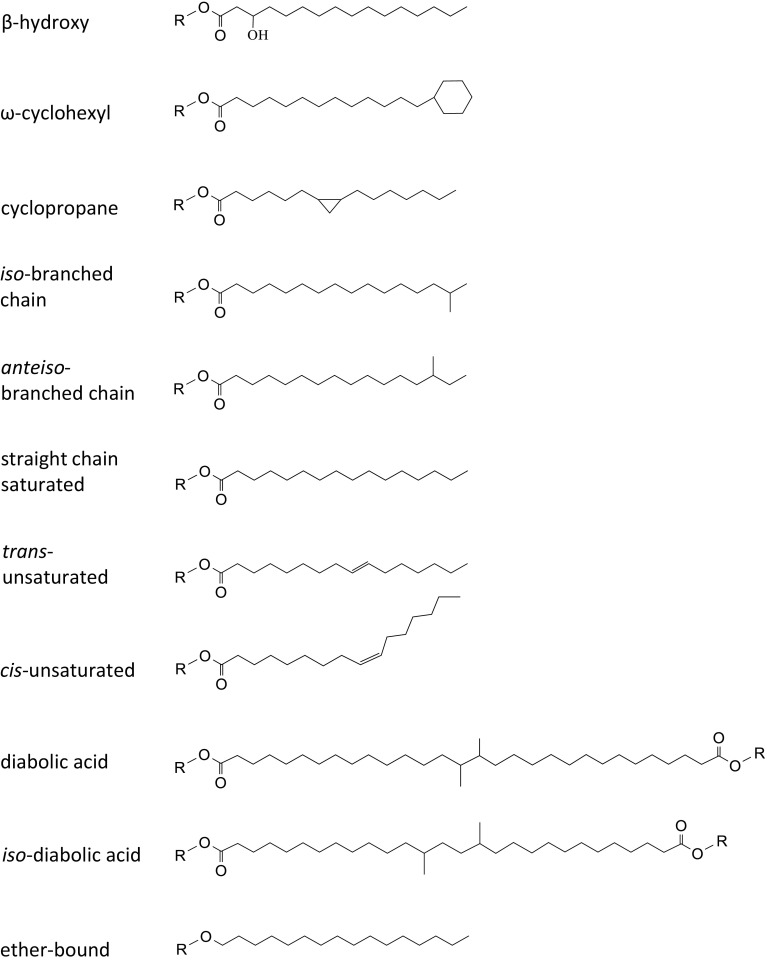
Variations in fatty acid chain conformation. *From top to bottom* common fatty acid modifications are depicted: β-hydroxy fatty acid, ω-cyclohexyl fatty acid, cyclopropane fatty acid, iso-branched chain fatty acid, anteiso-branched chain fatty acid, saturated straight chain fatty acid, trans-unsaturated fatty acid, cis-unsaturated fatty acid, diabolic acid, iso-diabolic acid, ether bound fatty acid. Adapted from (Chintalapati et al. 2004; Denich et al. 2003)

The environmental temperatures from which microbes are isolated roughly span from the freezing point of water and below for psychrophiles to the boiling point of water, for extreme hyperthermophiles. The challenges that microbes face below or above their optimal temperature is to retain optimal functionality of their macromolecules (nucleic acids, proteins, and lipids), aiming for a balance between stability (robustness) and flexibility (activity, transition states). As to bacterial membranes, the fluidity is dependent on the membrane’s phase-transition temperature (*T*_m_ or transition midpoint) that expresses the temperature at which a membrane shifts from the preferred liquid crystalline phase into the rigid gel phase when the temperature drops. More specifically, at the phase-transition temperature, 50% of hydrocarbon chains melt and a fluid and gel phase coincide. At temperatures below *T*_m_, lipids become ‘frozen’ by alignment of the hydrocarbon chains perpendicular to the plain of the bilayer (Eze 1991). This is a result of a close ordering and side-by-side packing of the immobilized hydrocarbon chains and gives rise to highly impermeable membranes. Not only is the barrier function affected, but many membrane proteins only function in the liquid crystalline phase (Russell 1990). Above *T*_m_, the phospholipid hydrocarbon chains are motile with a gradual increase of motility towards the core of the bilayer. The preferred liquid crystalline phase, therefore, provides a functional matrix for the many biochemical processes while being permeable to neutral molecules like H_2_O, CO_2_ and O_2_, but impermeable to ions and solutes (Konings et al. 2002; Mykytczuk et al. 2007). To maintain sufficient membrane fluidity below their optimal growth temperatures, bacteria adopt a large variety of modifications to lower the *T*_m_ (reviewed in (Chattopadhyay 2006; Chintalapati et al. 2004; Russell 1997; Shivaji and Prakash 2010)). The molecular mechanisms are directed at increasing the fluidity by forming a more disordered gel phase or by prevention of the gel phase. Distinct targets of bacterial cold adaptation (stress and physiological) have been identified: (i) unsaturated fatty acids (UFAs), (ii) short chain fatty acids (SCFAs), (iii) branched chain fatty acids (BCFAs), (iv) carotenoids, and (v) glycolipids and uncommon polar lipids. The most prevalent cold-stress modification is the incorporation of mono-unsaturated fatty acids (MUFAs) (Chintalapati et al. 2004; Russell 1997). Bacteria actively introduce cis- or trans-double bonds (Fig. 2) by desaturases or synthesize UFAs de novo (Suutari and Laakso 1994). The main advantage of implementing desaturases is the rapid response they elicit. The cis-unsaturated fatty acids, however, increase fluidity more efficiently than trans-unsaturated fatty acids. This is due to the immobile 30° kink in the acyl chain that increases the cross-sectional area of the lipid (Gruner et al. 1985).

The incorporation of cis-unsaturation is a well-described mechanism of cold-stress in the mesophile *Escherichia coli* (Marr and Ingraham 1962). Correspondingly, the opposite conversion of cis- to trans-unsaturated fatty acids has been correlated with adaptation to higher temperatures in *Pseudomonas syringae *(Kiran et al. 2004). The presence of poly-unsaturated fatty acids (PUFAs) in response to low temperatures is uncommon in mesophilic bacteria and far less effective in fluidization compared to mono-unsaturations. Nonetheless, omega-3 (ω3; EPA and DHA) and omega-6 (ω6; AA) PUFAs are abundantly detected in marine psychrophiles and cyanobacteria as an adaptation to low temperatures (Russell 1997; Shivaji and Prakash 2010; Zsiros et al. 2000). Fluidization of the membrane can also be achieved by the incorporation of SCFAs (<12 carbons), but can only be implemented in growing cells and are not employed as an abrupt stress adaptation (Denich et al. 2003). It is, therefore, commonly accepted that SCFA formation is not a universal way of fluidity modification during cold stress. Additional membrane adaptation strategies to sub-optimal temperatures have been observed in Gram-positive mesophiles (*Bacillus subtilis, Bacillus* T1) but also the Gram-negative thermophile *Thermus thermophilus,* in that they exchange iso- for anteiso-BCFA (Figs. 1b and 2) (Chintalapati et al. 2004; Oshima and Miyagawa 1974). This is because anteiso-positioned methyl groups cause greater fluidity of the membrane due to a greater disturbance of the packing order of the hydrocarbon chains. As with incorporation of SCFAs, BCFAs are synthesized de novo, and do not allow a swift response to sudden temperature drops. In the psychrotolerant *Sphingobacterium antarcticus* (*T*_opt_ = 25°) a combination of cold-specific modifications was detected (Jagannadham et al. 2000; Shivaji et al. 1992). When cultivated at 5 °C, the amount of UFAs is increased as well as the amount of BCFAs. Oppositely, at extremely low temperatures (i.e. −15 °C) the psychrotolerant species *Planococcus halocryophilus* (*T*_opt_ = 25 °C) exhibits a decrease in the amount of branched chain fatty acids emphasizing that the BCFA response is species- or situation-specific. In addition, polar and non-polar carotenoids (C40) were found to be incorporated into the membrane as shown for the psychrotolerant *Micrococcus roseus* (*T*_opt_ = 20 °C) (Chattopadhyay et al. 1997). These pigments are believed to insert their hydrophilic groups mainly at opposite sides of the polar regions of the bilayer and as such adopt a membrane spanning orientation (Gruszecki and Strzałka 2005). Ironically, polar carotenoids like zeaxanthins are believed to decrease membrane fluidity in the liquid crystalline phase, but increase fluidity in the gel phase. Polar carotenoids are, therefore, assumed to balance the fluidizing effect of the fatty acid modifications while simultaneously enhancing the barrier function to ions and oxygen (Chattopadhyay et al. 1997; Gruszecki and Strzałka 2005; Jagannadham et al. 2000).

**Fig. 3:**
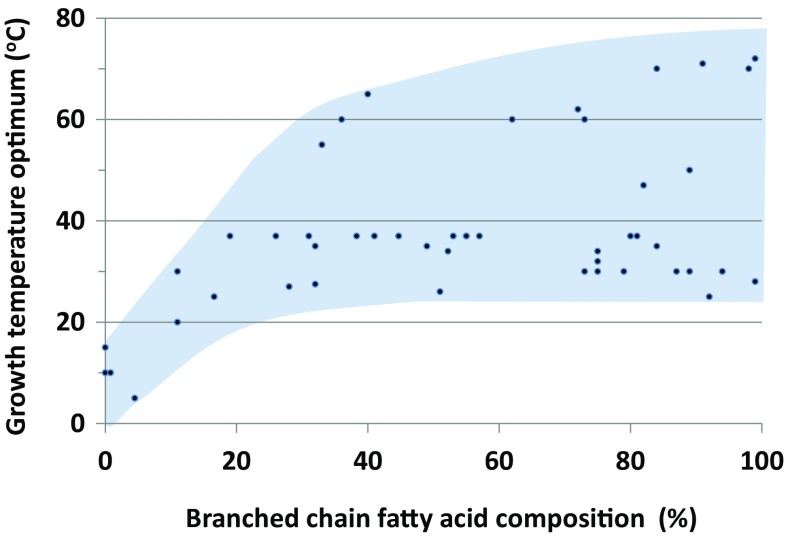
Bacterial growth temperature optima versus percentage BCFAs. Documented growth temperature optima of 48 bacteria plotted against the percentage of BCFAs of the respective bacterium. The trend shows that at low growth temperatures, BCFA percentages are generally low, while at moderate and high growth temperatures, BCFA percentages are variable

With respect to long-term physiological membrane adaptations to cold, many studies on the membrane lipid composition of psychrophilic bacteria (*T*_opt_ <15 °C) have been performed (Table 1). Membrane characterization of *Clostridium psychrophilum* (*T*_opt_ = 4 °C) (Guan et al. 2013; Spring et al. 2003), *Colwellia psychrerythraea* (*T*_opt_ = 8.5 °C) (Huston et al. 2004; Wan et al. 2016), and *Psychromonas ingrahamii* (*T*_opt_ = 5 °C) (Auman et al. 2006; Breezee et al. 2004) revealed comparable adaptations to cold. These adaptations also involve high levels of SCFAs, UFAs, polar carotenoids and glycolipids. Interestingly, although iso-BCFAs are detected in psychrophilic membranes, branching does not play a prominent role. Comparative analysis of BCFA percentages in bacterial psychrophiles and non-psychrophiles shows a negative relationship with growth temperature optima (Fig. 3; Online Resource 1). In *Psychromonas ingrahamii*, BCFAs levels of only 4.5% of the total fatty acid content are detected, and in *Colwellia psychrerythraea* or *Desulfotalea psychrophila* BCFAs are present at trace amounts or completely absent. This suggests that BCFAs confer no benefit and may even be disadvantageous at very low temperatures (*T*_opt_ ≤15 °C). When the psychrophilic *Clostridium psychrophilum* is cultivated below 0 °C, a combination of cold-specific modifications is detected like in *Sphingobacterium antarcticus*. These bacteria use a high degree of unsaturated, cyclopropane containing fatty acids and SCFAs. Additionally, polar lipid changes are represented by a high degree of glycolipids, sn1-ether fatty acid plasmalogens and cardiolipins (CLs). These comparisons indicate that long-term physiological adaptations comprise highly similar mechanisms compared to the short-term stress adaptations, except for the contribution of BCFAs.

**Table 1:** Physiological membrane adaptations by core lipid modifications typically found in bacterial and archaeal extremophiles

Bacteria	Temperature					pH				Pressure	
	*T*_min_ <15 °C			*T*_max_ >75 °C		pH_min_ <3		pH_max_ >10		>70 MPa	
Level of chain length			Ref		Ref		Ref		Ref		Ref
shorter chain ≤C14	+		(7,8)			+	(31, 33)	+	(42, 43)		
longer chain ≥C18											
Level of unsaturation											
PUFA	+		(1–3)							+	(39)
MUFA-cis	+		(7,8, 40)	+	(21)	+	(33)	+	(44)		
MUFA-trans	+		(8)								
Level of branching											
BCFA-iso				+	(4,15,41)	+	(29)	+	(38, 42–44)		
BCFA-*anteiso*				+	(4)	+	(29, 32)	+	(44)		
Diabolic acid				+	(18, 45)	+	(35)				
(β)-hydroxy FA	+		(8)			+	(30, 33)				
Level of cyclization											
Ω-Cyclohexyl						+	(29, 32)				
Cyclopropyl	+		(7)	+	(21)	+	(30, 33)				
Level of tetraester and etherlipids											
Tetraesters				+	(22, 46)						
Mono- di- tetraethers				+	(18–21)	+	(34, 35)				
Level of terpenes											
Polar carotenoid	+		(5,6)	+	(16, 17)						
Non-polar terpenes								+	(44)		
Other modifications											
Cardiolipins	+		(7)					+	(44)		
Glycolipids	+		(7)	+	(16)						
BMP								+	(44)		

Archaea	Temperature				pH				Pressure	
	*T*_min_ <15 °C		*T*_max_ >75 °C		pH_min_ <3		pH_max_ >10		>40 MPa	
Level of chain length		Ref		Ref		Ref		Ref		Ref
C20-chain	+	(9)	+	(24–26)			+	(47–53)	+	(28, 54–55)
C25-chain			+	(56)			+	(47–53)		
Level of saturation										
Unsaturated diethers	+	(9, 10)	+	(11)						
Level of branching										
Hydroxyarchaeol	+	(9)								
Level of cyclization										
Pentacyclic TE			+	(13, 27)	+	(13,27, 36, 37)				
Macrocyclic			+	(57)					+	(28, 57)
Level of tetraether lipids										
Tetraethers	–	(9)	+	(12,23)	+	(14,36, 60)	–	(61, 62, 63)	–	(28)
Other modifications										
Glycolipids			+	(11)	+	(27, 37)	–	(48, 50, 53, 58, 59)		

References: 1: Russell (1997), 2: Shivaji and Prakash (2010), 3: Zsiros et al. (2000), 4: Oshima and Miyagawa (1974), 5: Chattopadhyay et al. (1997), 6: Jagannadham et al. (2000), 7: Guan et al. (2013), 8: Wan et al. (2016), 9: Nichols et al. (2004), 10: Gibson et al. (2005), 11: Sprott et al. (1997), 12: Cario et al. (2015), 13: De Rosa et al. (1980), 14: Uda et al. (2004), 15: Patel et al. (1991), 16: Ray et al. (1971), 17: Yokoyama et al. (1996), 18: Damsté et al. (2007), 19: Langworthy et al. (1983), 20: Huber et al. (1992), 21: Jahnke et al. (2001), 22: Huber et al. (1989), 23: Matsuno et al. (2009), 24: De Rosa et al. (1987), 25: Hafenbradl et al. (1996), 26: Ulrih et al. (2009), 27: Schleper et al. (1995), 28: Kaneshiro and Clark (1995), 29: De Rosa et al. (1974), 30: Wichlacz et al. (1986), 31: Wakao et al. (1994), 32: Matsubara et al. (2002), 33: Mykytczuk et al. (2010), 34: Weijers et al. (2006), 35: Damste et al. (2011), 36: Macalady et al. (2004), 37: Uda et al. (2001), 38: Clejan et al. (1986), 39: Nogi and Kato (1999), 40: Knoblauch et al. (1999), 41: Heinen et al. (1970), 42: Li et al. (1994), 43: Prowe and Antranikian (2001), 44: Clejan et al. (1986), 45: Balk et al. (2009), 46: Lee et al. (2002), 47: Namwong et al. (2007), 48: Feng et al. (2005), 49: Castillo et al. (2006), 50: Xu et al. (2001), 51: Xu et al. (1999), 52: Lanzotti et al. (1989), 53: Tindall et al. (1984), 54: Grant et al. (1985), 55: Takai et al. (2000), 56: Sako et al. (1996), 57: Sprott et al. (1991), 58: Hu et al. (2008), 59: Bowers and Wiegel (2011), 60: Schleper et al. (1995), 61: Feng et al. (2005), 62: Xu et al. (2001), 63: Lanzotti et al. (1989)

*PUFA* polyunsaturated fatty acids, *MUFA-cis* cis-monounsaturated fatty acids, *MUFA-trans* trans-monounsaturated fatty acids, *BCFA-iso* iso-branched chain fatty acids, *BCFA-anteiso* anteiso-branched chain fatty acids, *BMP* bis-mono-acylglycero-phosphate, *TE* tetraethers, *+* increased production, − decreased production

### Low temperature adaptation in archaea

**Fig. 4:**
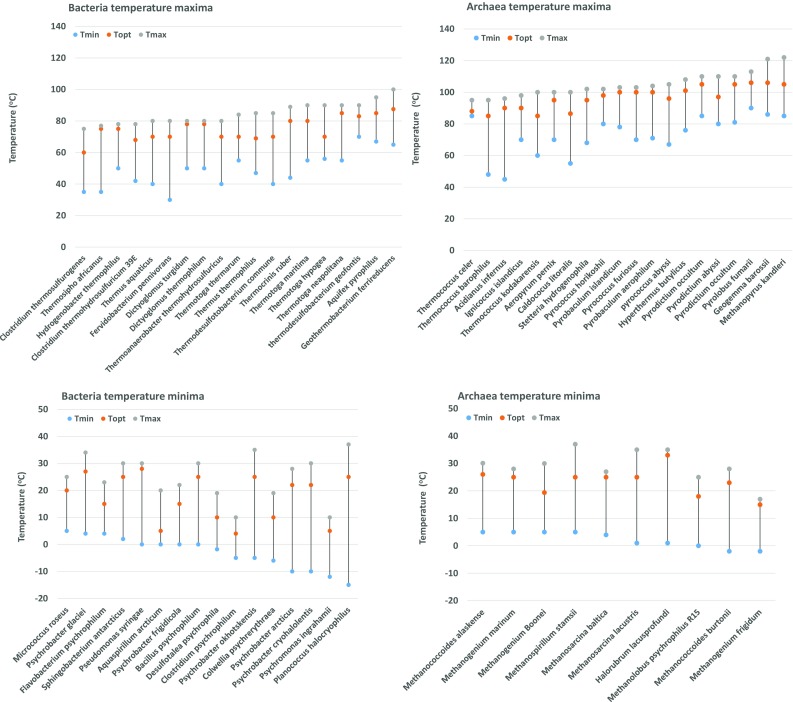
Reported maximal, optimal and minimal growth temperatures of presently studied most extreme hyperthermophiles and psychrophiles. Hyperthermophilic bacteria and archaea are depicted in the *upper panels* and grouped according to increasing maximal growth temperature. Psychrophilic bacteria and archaea are depicted in the lower panels and grouped according to decreasing minimal growth temperature

Membrane adaptation to cold has not been studied as extensively in archaea as for bacteria. Cold-adaption in archaea was long time overlooked probably due to the late recognition of their abundance in oceans and a main interest for hyperthermophilic archaea instead. Nonetheless, it appears that bacterial psychrophiles outnumber archaea in diversity and dominate subfreezing ecosystems (Fig. 4 lower panels) (D’Amico et al. 2006). According to the standard terminology of psychrophilicity, (*T*_opt_ ≤15 °C, i.e. growth optimum at or below 15 °C), there are at present only 2 confirmed species of archaeal psychrophiles [*Cenarchaeum symbiosum T*_opt_ = 10 °C (Preston et al. 1996) and *Methanogenium frigidum T*_opt_ = 15 °C (Franzmann et al. 1997)]. Here we will, therefore, apply an adjusted terminology for archaeal psychrophilicity (T_min_ < 5 °C, i.e. capable of growth below 15 °C). *Methanosarcinaceae* and *Methanomicrobiales* are thus far the most studied archaeal psychrophiles, but in-depth analyses of physiological membrane adaptations are lacking. The few reported lipid adaptation studies were stress response studies and demonstrate a couple of mechanisms to increase membrane fluidity with only the incorporation of unsaturations being analogous to bacteria. The documented stress responses to cold are collectively grouped as following: (i) unsaturated diethers, (ii) isoprenoid hydroxylation, (iii) tetraether:diether ratio, and (iv) number of pentacycli. In *Methanococcoides burtonii* (T_opt_ = 23 °C) (Nichols et al. 2004) and *Halorubrum lacusprofundi* (*T*_opt_ = 33 °C) (Gibson et al. 2005), membranes are found that completely lack GDGTs and have an increased level of unsaturated diether lipids. The biosynthesis of unsaturated archaeol is probably a passive event in which the fully unsaturated precursor DGGGP is selectively or incompletely saturated by reductases, unlike the active introduction of double bonds by desaturases typical for bacteria. Unsaturated archaeol, however, was also found in the hyperthermophile *Methanopyrus kandleri* (Sprott et al. 1997), currently the record-holder of highest maximum growth temperature. This discrepancy seriously questions both the fluidizing effect and the absolute requirement of unsaturated diethers as psychrophilic adaptation mechanism in archaea. In addition to high levels of unsaturated archaeol, a significant amount of hydroxyarchaeol was also observed in *Methanococcoides burtonii* (Nichols et al. 2004). This modification involves the hydroxylation at the C-3 position of the sn-1 isoprenoid chain. The authors suggest that this modification results in an extension of the polarity of the head group, thereby shortening the core of the lipid. It is, however, not certain whether this type of adaptation is really cold-specific. Although archaea are also known to intercalate non-polar poly-isoprenoids like lycopene and squalene between their membrane lipids, it is not demonstrated to result in fluidity buffering like polar carotenoids do in bacterial membranes. In the extreme halophile *Halobacterium salinarum*, squalene is detected at high quantities and shown to play a major role in packing and lateral organization of the polar lipids (Gilmore et al. 2013). A role of squalene in fluidity adaptation, comparable to carotenoids in bacteria, is therefore, also expected in psychrophilic archaea. Squalene is probably implemented to rigidify and reduce permeability to protons and solutes.

Archaeal cold stress adaptation studies are mostly done on thermophiles grown below their optima. From these archaeal below-optimum adaptation studies a recurring theme is an increase in diether content and resulting decrease of membrane spanning GDGTs (Fig. 1g, h). In *Thermococcus kodakarensis* (*T*_opt_ = 85 °C), a temperature drop from 85 to 60 °C causes archaeol (DE) to increase from 17.7 to 49.1% at the expense of GDGTs (82.3 to 50.9%) (Matsuno et al. 2009). Correspondingly, for *Archaeoglobus fulgidus* (*T*_opt_ = 78 °C) the diether content shifts from 60 to 70% when grown at a relatively low temperature of 70 °C (Lai et al. 2008). One of the most recently described below-optimum-stress adaptation in archaea concerns a decrease in pentacycle number. Archaeal pentacyclization is a modification initially observed in hyperthermophiles. This feature involves the incorporation of cyclopentane rings along the biphytanyl chains up to 4 rings per chain (Fig. 1h). These rings are believed to stabilize chain packaging and to decrease membrane permeability. In *Thermoplasma acidophilum* (*T*_opt_ = 59 °C), lowering the growth temperature to 40 °C results in a decreased average number of pentacycles per lipid. Here, a change in cyclization degree from 2.1 to 1.6 cycles per lipid was observed (Uda et al. 2001). A decrease in tetraether and pentacycle number, however, should not be regarded as universal cold-shock response. This is because membranes that are exclusively composed of tetraethers with high numbers of pentacycles per lipid have also been reported to be common in mesophilic archaea (Oger and Cario 2013). Drawing general conclusions from these changes is thus hampered by the fact that most of these studies are based on hyperthermophiles only. Moreover, the observed temperature effects should be normalized by growth phase, which has not always been done.

Due to the lack of reported archaea growing below −2 °C, it is tempting to suggest that bacteria are better equipped to growth in extremely cold habitats. Although this can be due to a sampling and cultivation bias, this hypothesis is supported by recent meta-genomics and transcriptomics sequence analysis of the accretion ice of subglacial lake Vostok (Antarctica) (Shtarkman et al. 2013). Here, the metagenome showed a distribution of 94% bacterial sequences, 6% eukaryal and only a small number of archaeal sequences. Additionally, there is a difference in lowest reported growth temperature minima between archaea and bacteria (Fig. 4 lower panels). The lowest confirmed growth optimum for archaea lies at 15 °C for *Methanogenium frigidum* (Franzmann et al. 1997). This is notably higher than the optimum of 5 °C for the bacterium *Psychromonas ingrahamii*, whose lowest demonstrated growth temperature lies at a startling −12 °C (Auman et al. 2006). In case there is truly a growth temperature boundary for archaea around the freezing point of water, a plausible explanation for this threshold may lie in the methyl branched hydrocarbon chains. In the comparative analysis of BCFA percentages we showed that psychrophilic bacteria generally have less methyl branched lipids compared to mesophiles and thermophiles suggesting a disadvantage in cold environments. Methyl branched phytanyls on the other hand, are an imperative feature of archaeol. The isoprenoid constituent of archaeal membranes may, therefore, restrict efficient adaptation mechanisms required to maintain fluidity at subfreezing temperatures. This apparent difference observed in growth temperature minima between archaea and bacteria also leads to interesting questions concerning lipid phases and functionality of the archaeal membrane, because the *T*_m_ of some archaeal membranes is established at −15/−20 °C or even lower (Blocher et al. 1990; Dannenmuller et al. 2000; Koga 2012). This average *T*_m_ thus suggests that archaeal membranes can maintain a liquid crystalline phase below 0 °C without the requirement of extensive fluidity enhancing modifications. Apparently, the isolated psychrophilic archaea and their established growth minima so far do not support this and indicate that other factors than membrane fluidity become limiting for archaea at low temperature.

### High temperature adaptation in bacteria

At higher temperatures, microbes endure increased fluidity and permeability of the membrane up to a point that lipids pack too disordered to maintain a liquid crystalline phase. When the temperature rises beyond the optimum of the liquid-crystalline phase, lipids adopt a fluid phase and ultimately also a non-lamellar phase like the cubic and hexagonal structure (Escriba 2006). It is, therefore, not surprising to find adaptation strategies which are opposite to cold adaptations, like those that induce stiffening and promote ordering of the hydrocarbon chains. Mesophilic and thermophilic bacteria mainly adjust fluidity by increasing the amount of (i) branched chain iso-fatty acids (Patel et al. 1991; Sinensky 1971, 1974), (ii) saturated fatty acids (Oshima and Miyagawa 1974), (iii) long-chain fatty acids and (iv) polar carotenoid content (Ray et al. 1971; Yokoyama et al. 1996). Counterintuitively, the membranes of thermophilic bacteria are not devoid of cis-monounsaturated and anteiso-BCFAs (Patel et al. 1991) which are typically implemented as cold stress adaptation. What appears more important is the ratio between these fatty acids. In the highest temperature window of bacterial hyperthermophiles, some bacteria amend high amounts of BCFAs, which occasionally approach 100% of the fatty acids (Fig. 3). These BCFAs are for the greatest part composed of iso-BCFAs. Interestingly, thermophilic bacteria that are able to grow above 70 °C, implement lipid species that more or less remind of typical archaeal lipids (tetraethers, diethers and tetraesters). A key question that then arises is whether these lipids are typical physiological adaptations to heat, or whether they are remnants of these bacteria phylogenetically deeply rooted position. In *Thermotogales* species, e.g. (*T*_max_ = 90 °C) diabolic-acid derived membrane spanning tetraether-lipids are detected (Damsté et al. 2007). Diether fatty acid lipids are found in *Thermodesulfotobacterium commune T*_max_ = 85 °C (Langworthy et al. 1983) and *Aquifex pyrophilus T*_max_ = 95 °C (Huber et al. 1992; Jahnke et al. 2001). Lastly, long chain dicarboxylic fatty acid dimethyl-esters or in short, tetraester-lipids are found in *Thermoanaerobacter ethanolicus T*_max_ = 78 °C (Jung et al. 1994; Sanghoo Lee et al. 2002), *Thermoanaerobacter thermosulfurigenes T*_max_ = 75 °C (Russell and Fukunaga 1990), *Thermosipho africanus T*_max_ = 77 °C (Huber et al. 1989), *Fervidobacterium pennivorans T*_max_ = 80 °C (Damsté et al. 2007) and more. Analogous to archaeal tetraether lipids, these bacterial tetraether and tetraester lipids are believed to result from a tail-to-tail condensation between two ‘regular’ iso-branched chain fatty acids that extend from both leaflets of the membrane. Contrary to the earlier dogma, these mentioned bacterial species show that membrane spanning hydrocarbon chains are not an absolute requirement for survival beyond 80 °C. This becomes especially apparent by *Aquifex pyrophilus* (*T*_max_ = 95 °C) that does not have membrane spanning lipids but does contain a high amount of ether lipids. It must be emphasized here that in this species also a minority of ester-lipids was isolated, which also shows the ability of ester lipids to tolerate these temperatures in vivo. Nonetheless, this analysis points at a temperature-dependent boundary at ~80 °C for bacterial membranes exclusively composed of ester-lipids. This is in line with the postulation of Robert Huber and Karl Stetter that ether bonds are essential for hyperthermophilic growth (Huber et al. 1992).

### High temperature adaptation in archaea

Currently, the highest reported growth maximum for bacteria was demonstrated for *Geothermobacterium ferrireducens* (*T*_max_ = 100 °C) (Kashefi et al. 2002) as opposed to the archaeal record holder *Methanopyrus kandleri* (*T*_max_ = 122 °C) (Takai et al. 2008) (Fig. 4 upper panels). For thermophilic archaea, membrane spanning tetraether lipids are the most abundant and frequently also the only core lipid. Besides this regularity, not much variability is observed in hyperthermophilic membranes. Tetraether lipids form monolayers that are highly stable due to a restricted motility of the hydrocarbon chains. Raising the growth temperature, therefore, elicits an increased tetraether/diether ratio like shown for *Thermococci* (Cario et al. 2015; Matsuno et al. 2009). Presence of tetraethers thus appears to be highly supportive for hyperthermophilic growth, a rule that also became apparent in extreme hyperthermophilic bacteria which sometimes also use tetraether lipids. A tetraether-containing membrane, however, might not be a prerequisite for heat tolerance, as *Methanopyrus kandleri,* and *Aeropyrum pernix* were reported to have no or only trace amounts of tetraethers but grow optimally between 95 and 105 °C (Hafenbradl et al. 1996; Morii et al. 1999; Sprott et al. 1997; Ulrih et al. 2009). The already high stability of tetraether membranes can be further increased by the incorporation of pentacycli that cause an up-shift of the transition temperature (Gliozzi et al. 1983). In line with this, an increase in the number of pentacycli per lipid is frequently observed, like in *Thermoplasma acidophilum* and *Sulfolobus solfataricus* (De Rosa et al. 1980; Uda et al. 2001). Finally, heat adapted membranes may involve macrocyclic archaeols (Kaneshiro and Clark 1995). In these modified archaeols the isoprenoid chains are cross-linked at their tail-ends, and like tetraethers dramatically restricted in their motion, causing an improved membrane barrier to water and membrane stability (Fig. 1f) (Dannenmuller et al. 2000).

Although the various reported lipid modifications suggests certain overall trends in membrane adaptations to high temperature, several exceptions to these rules have been reported as well. By far the most invalidating finding might be the observed minority of GDGTs in the membrane of the hyperthermophile *Methanopyrus kandleri* (*T*_max_ = 122 °C) (Hafenbradl et al. 1993; Kurr et al. 1991; Sprott et al. 1997; Takai et al. 2008). However, it must be stressed here that initially also for *Thermococcus barophilus* and *Thermococcus celer* no GDGT’s were demonstrated, but upon re-evaluation the lack was shown to be an artefact of the extraction method used (Cario et al. 2015; Sugai et al. 2004). The analysis of intact polar lipids (IPL’s) with the omission of acid hydrolysis has shown to cause major erroneous membrane compositions. Conflicting data was also observed for glycolipids, that were found at high levels, but also low levels under high temperature conditions (De Rosa et al. 1987). The implementation of ether-bonds seems to form a high correlation with thermal adaptation in bacteria that generally do not use ether-bonds. Despite the exceptions, the boundary of survival for bacteria appears to be around 100 °C. Altogether, the general trends indicate that bacterial and archaeal membrane adaptations to temperature are complex and make use of different strategies (Table 1). However, the question then remains what differentiates the bacteria from the archaea with respect to adaptations to low and high temperatures. Bacteria maintain a proper level of permeability and acceptable fluidity at temperatures only just above their phase transition temperatures. Permeability and fluidity of archaeal membranes on the other hand generally stay optimal while the membrane remains in the liquid crystalline phase throughout the entire biological temperature span between 0 and 100 °C. Oftentimes, it is therefore, hypothesized that the archaeal biphytanyl hydrocarbon bonds linked to glycerol by ether bonds confer higher thermal stability, increased rigidity and reduced permeability to the archaeal membranes (Koga 2012; Mathai et al. 2001). This may explain why some archaeal membranes do not extensively modify their core lipids. Our overall comparison of the lipid compositions also suggest that a lower number of ether bonds and level of branching highly determine the lower growth temperature boundary of thermophilic bacteria compared to archaea. What remains elusive in archaeal membrane adaptation to temperature, however, is what the optimal phase for membrane functionality is, and to what extent glycolipids and how polar heads are involved in this regulation. A study on liposome phase behaviour in *Sulfolobus acidocaldarius* showed the coexistence of multiple phases around the growth temperature optimum, and more recently large variations in polar heads were detected in response to temperature fluctuation in *T. kodakarensis*. More general insight in archaeal phase transitions and the role of polar heads are thus essential to provide clarity in this issue.

### High pressure adaptation in bacteria and archaea

**Fig. 5:**
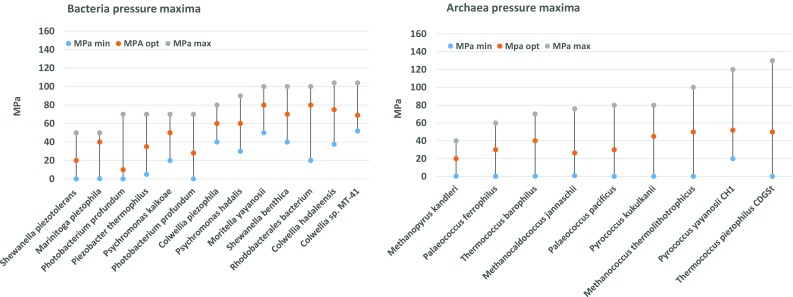
Reported maximal, optimal and minimal pressure-values of presently studied most extreme piezophiles/barophiles. Barophilic bacteria and archaea are grouped according to increasing maximal pressure tolerance

In deep-sea marine environments, microorganisms are exposed to high hydrostatic pressure. These pressures also affect the membrane fluidity, and membranes are even labelled as the most pressure-sensitive biological structures (Oger and Jebbar 2010). At increasing pressures, lipids pack more tightly and membranes consequently loose fluidity, permeability and enter the gel phase similar to cold conditions (Casadei et al. 2002). Deep-sea piezophiles/barophiles are found to grow and even require pressures up to maximally 120 MPa (Yayanos 1986; Zeng et al. 2009). For piezophilic bacteria and archaea, a slight difference is observed between their survival boundaries, although this is only based on studies of the bacterium *Colwellia* sp. MT-41(pressure_max_ = 103 MPa)(Yayanos et al. 1981) and of the archaeon Thermococcus piezophilus (pressure_max_ = 130 MPa) (Dalmasso et al. 2016) (Fig. 5). The main difference of piezophilic from psychrophilic adaptation in bacteria is the prominent presence of PUFAs (Bartlett 1992) in addition to MUFAs. In *Alteromonas* sp. the amount of long chain PUFA 20:5 has been shown to increase with pressure (Wirsen et al. 1987). In contrast, it was shown in *Photobacterium profundum* that the MUFAs and not the PUFAs are correlated with survival at high pressure in bacteria (Allen et al. 1999). The accumulation of the polar lipids phosphatidylglycerol and phosphatidylcholine instead of phosphatidylethanolamine is also a frequently observed adaptation (Jebbar et al. 2015; Mangelsdorf et al. 2005; Yano et al. 1998). It is generally accepted that larger head groups lead to greater disruption of membrane packing and hence to enhance membrane fluidity (Jebbar et al. 2015; Mangelsdorf et al. 2005). Studies performed with archaea on the homeoviscous adaptation to pressure are scarce and likely hampered by the fact that the identified piezophilic archaea were frequently also (hyper) thermophiles. Currently, only two studies elaborately studied the effect of increased pressure on the membrane composition of archaea. In *Methanococcus jannaschii*, an increase in the macrocyclic archaeol and concomitant decrease in archaeol and GDGTs has been observed (Kaneshiro and Clark 1995). The difficulty of studying the sole effect of pressure on archaeal membranes is exemplified in a study on the piezophilic hyperthermophilic *Thermococcus barophilus*. Here a similar decrease of GDGTs and concomitant increase in archaeol was found which completely resembles cold adaptation (Cario et al. 2015). Interestingly, these cocci show an adapted level of unsaturation of apolar lipids (Cario et al. 2015), but it is left unclear whether this is a temperature or pressure induced effect. The common feature, however, with bacteria is that increased pressure leads to adaptations that result in more fluid membranes.

### Low pH adaptation in bacteria

Like the discovery of microorganisms thriving at extreme temperatures, also bacteria and archaea have been found in ecosystems with extreme pH values. Bacteria and archaea have evolved various ways to cope with extreme acidity (<pH 2) or alkalinity (>pH 10). The challenge both acidophiles and alkaliphiles are facing is to retain a near neutral intracellular pH. The ΔpH (pH_in_–pH_out_) across the membrane is a major component of the proton motive force (PMF), and as such important for the energy supply of the cell. The cells actively pump out protons by means of a respiratory chain or photosystem. This outflux generates a proton motive force (PMF) that is used by ATPases to generate chemical energy in the form of ATP. Upon external down-shifts in pH, the PMF needs to be adjusted by a higher activity of the respiratory chain or other proton-pumping systems. Acidophiles evolved several mechanisms to maintain near neutral intracellular pH. They, for example, reverse their membrane potential (Δψ) to deflect intrusion of protons. A higher expression of proton exporters and secondary transporters is therefore, a common strategy. Often, enzymes and chemicals are produced that bind or buffer protons. The most effective strategy, however, lies in reduction of proton permeability by the plasma membrane. The neutralophilic bacterium *Escherichia coli*, remodels its membrane, based on only three strategies: (i) an increase in short straight-chain fatty acids content, (ii) a decrease in the amount of unsaturations and (iii) lowering the amount of cyclopropane-fatty acids (Yuk and Marshall 2004) (Fig. 2). These modifications, however, do not follow a consistent pattern with other pH stress studies on neutralophiles. A decrease in short-chain fatty acids was observed in *Streptococcus mutans* instead (Fozo and Quivey 2004). In this species, another opposite effect was also observed for the number of unsaturations, which showed an increase to acid stress. Another quite contrasting finding was the fact that a cyclopropane fatty acid knockout in *Salmonella enterica typhimurium* showed a sensitivity to low pH, suggesting a positive relation with these fatty acids instead (Kim et al. 2005). This sensitivity to low pH was also observed in an *Escherichia coli cfa* knock-out strain (Chang and Cronan 1999). As a fourth adaptation to low pH values an overall increase of anteiso- and simultaneous decrease in iso-BCFAs was observed in *Listeria monocytogenes* (Giotis et al. 2007).

**Fig. 6:**
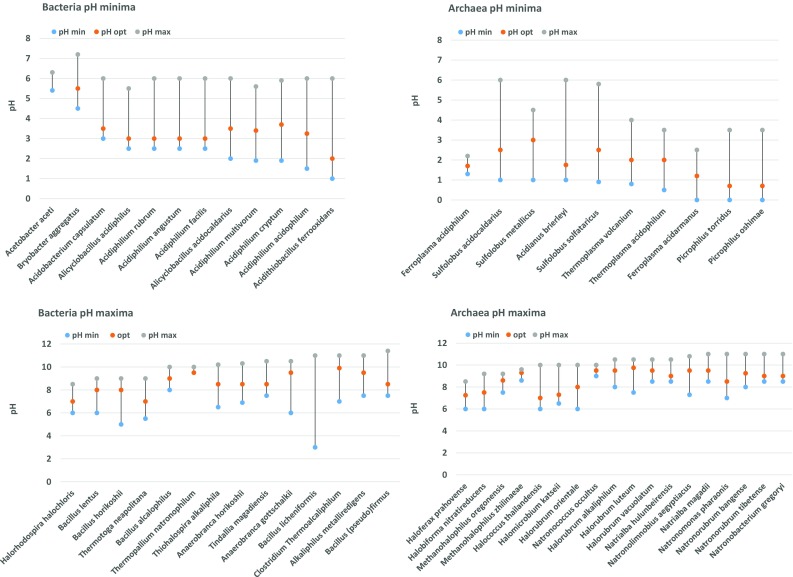
Reported maximal, optimal and minimal pH-values of presently studied most extreme acidophiles and alkaliphiles. Acidophilic bacteria and archaea are depicted in the *upper panels* and grouped according to decreasing minimal pH values. Alkaliphilic bacteria and archaea are depicted in the *lower panels* and grouped according to increasing maximal pH values

Obligate acidophiles synthesize a membrane that is highly impermeable to protons (Baker-Austin and Dopson 2007). More consistent physiological membrane adaptations are described in extreme acidophilic bacteria of which *Alicyclobacillus* (*Bacillus*) *acidocaldarius* (De Rosa et al. 1974), *Acidiphilium* sp. (Wakao et al. 1994; Wichlacz et al. 1986), *Alicyclobacillus acidiphilus* (Matsubara et al. 2002) and *Acidithiobacillus ferro*-*oxidans* (Mykytczuk et al. 2010) are most thoroughly studied. These membranes are composed of high levels of iso- and anteiso-BCFAs, both saturated and mono-unsaturated fatty acids and uncommon β-hydroxy-, ω-cyclohexyl (Fig. 2) and cyclopropane fatty acids. These permanent modifications were detected in the membranes of acidophilic bacteria which tolerate growth at pH ≤2.5. In peat bogs (pH 3–5) GDGT-like lipids with branched dicarboxylic fatty acids (iso-diabolic acid) bound by ether-bonds to G-3-P have been detected (Weijers et al. 2006). These lipids, that highly resemble the archaeal membrane spanning lipids, are clearly of bacterial origin and seem to correlate with the low pH of the habitat from which they were isolated. A widespread presence of the iso-diabolic acid lipids was also detected in various species of the phylum *Acidobacteria*, but do not grow below pH 3 (Damste et al. 2011). Here we find another example for the usage of archaeal-like lipids implemented by bacteria, possibly to deal with the environmental stress. Despite their numerous adaptation strategies, bacteria have not been demonstrated to grow at pH <1 as opposed to archaea (Fig. 6 upper panels).

### Low pH adaptation in archaea

As with temperature adaptations, the three typical homeoviscous adaptation strategies of archaea are also observed in acidic environments. A key characteristic of acidophilic archaea is the presence of a membrane monolayer typically composed of nearly 100% GDGTs (Macalady et al. 2004; Oger and Cario 2013). Biophysical studies on liposomes composed of GDGTs showed that monolayer membranes are extremely impermeable to protons (Elferink et al. 1994). Extreme acidophilic archaea, able to thrive at pH <1, are also shown to incorporate multiple cyclopentane rings, to enhance lipid packing, compressibility and membrane rigidity even more (De Rosa et al. 1980; Macalady et al. 2004; Schleper et al. 1995; Uda et al. 2001). Whether the number of cyclopentane rings is really correlated with acidophilicity, however, is still a matter of debate. Nonetheless is it highly supported by a molecular modelling study that showed a tighter packed structure compared to GDGTs without rings (Gabriel and Chong 2000). The relation of cyclopentane rings with thermophilicity is shown multiple times, but in the case of acidophiles hampered by the fact that these species are often also thermophiles. The number of cyclopentane rings, e.g. was shown to decrease in *Thermoplasma acidophilum* when grown at lower pH values (Shimada et al. 2008). In contrast, *Acidilobus sulfurireducens* exhibits an increase in the cyclopentane rings at low pH values (Boyd et al. 2011). Apparently, the pH stress response shows a species- or temperature-dependent response in terms of the number of cyclopentane rings. A study on the extreme acidophile *Picrophilus oshimae* (pH_opt_ = 0.7) may hint on the cause of this discrepancy. When the lipids were extracted and re-constituted to liposomes they were unable to assemble when suspended at a pH >4.0 (van de Vossenberg et al. 1998a, b). As this finding highly contradicts the self-assembly capabilities of archaeal lipids from neutralophilic hyperthermophiles with the same core-lipid architecture, it suggests a putative important role for polar head-groups in physiological membrane adaptations of acidophiles and perhaps also for low pH stress response. Here, repulsive charges of the polar head groups at high pH could be the explanation for reduced membrane packaging of the lipids. Nonetheless, membrane spanning lipids have been shown to be correlated with acidophilic archaea (Macalady et al. 2004). Because bacteria are also known to generate membrane spanning lipids, the difference in lower pH boundary of archaea can be attributed to cyclopentane rings and the abundant methyl-branches. Although it is evident that these features lend archaea a lower permeability compared to standard bacterial lipids, the membrane spanning lipids in bacteria do not enable them to survive equally low pH levels as archaea. In a molecular dynamics study, (Chugunov et al. 2014) simulated the effect of methyl-groups and cyclopentane rings on membrane permeability and fluidity. A prominent finding was that the presence of methyl groups confers greater fluidization and higher permeability to the membrane than membrane spanning lipids without methyl groups. It is very likely that the inability of bacteria to include multiple methyl chains in their hydrocarbon chains explains why membrane spanning lipids are not detected in the most extreme acidophilic bacteria (pH_min_ <3).

### High pH adaptation in bacteria and archaea

On the other side of the pH scale, we encounter the obligate alkaliphiles. In sharp contrast to acidophiles, bacteria survive at comparable and even slightly higher pH values than archaea, with ~pH = 11.4 as the upper limit for bacteria (Fig. 6 lower panels). This suggests that the adaptation mechanisms of archaea and bacteria to high pH are nearly equivalent in efficiency. Although studies on the membrane composition in alkaliphilic bacteria are scarce, a few general trends are observed. The first striking observation is the high variability of lipids present in alkaliphilic bacteria. Often specific species like bis-mono-acylglycero-phosphate (BMP) lipids and CLs are detected in high quantities (Krulwich 2006).The alkaliphilic membranes are also copiously enriched in BCFAs (both iso as anteiso), and oftentimes MUFAs are very abundant (Clejan et al. 1986; Li et al. 1994; Rainey et al. 1996; Ye et al. 2004). The actual upper pH limit for life was described for a bacterium, *Bacillus pseudofirmus* (pH_max_ = 11.4) (Janto et al. 2011). In this species, high levels of CL, BMP, squalenes, and carotenoids were detected. Furthermore, the fatty acids were mainly composed of MUFAs and 92% BCFAs (Clejan et al. 1986). Altogether, it is concluded that enrichment of BCFAs is a recurring theme in extremophilic bacteria in general, except for the psychrophiles. Another alkaliphilic characteristic is the presence of squalenes, tetrahydrosqualenes or other polyisoprenes in the membrane. Hauß et al. showed that these neutral lipids specifically position in the centre of the lipid bilayer, parallel to the plane of the membrane (Hauß et al. 2002), where they are thought to reduce lipid motility and proton leakiness as proposed by (Haines 2001). Furthermore, water permeability is reduced and membrane rigidity increased which is an adaptation mechanism also suitable for low pH or high temperature. Squalene incorporation is also one of few adaptation mechanisms that are shared by both archaea and bacteria. In alkaliphilic archaea one can recognize a very distinct pattern from other archaea in which tetraethers and its derivatives are completely absent. Alkaliphilic archaeal membranes are dominated by diether lipids composed of C20:C20 or C20:C25 core lipids (Oger and Cario 2013) and a complete absence of GDGTs. The same holds true for glycolipids which are found only in trace amounts or which are completely absent. The polar headgroups are typically dominated by phosphatidylglycerol and phosphatidyl-glycerol phosphate methyl ester (PGP-Me) causing an overall negative charge of the membrane that strongly influences permeability to protons and water.

### Concluding remarks on extremophile membrane adaptations

Various studies have shown that archaeal membranes have more robust properties compared to bacterial membranes (Koga and Morii 2005; Mathai et al. 2001; van de Vossenberg et al. 1998a, b; Yamauchi et al. 1993). Nevertheless, both archaea and bacteria inhabit extreme environments as extensively as mild environments. To provide insight into the occurrence of both domains in extreme environments we made an inventory of maximal and minimal growth conditions of well characterized hyperthermophiles, psychrophiles. This analysis demonstrates an equally broad temperature growth range of ~120 degrees for both archaea and bacteria. An important difference, however, lies in the domination at the temperature extremes. Bacteria are found to dominate lower temperature ecosystems, whereas archaea dominate the higher temperature ecosystems. A comparable pattern also appeared when we analysed the pH scale where archaea dominate the bacteria <pH 1, and bacteria show a slight benefit at high alkalinity >pH 11. Regardless of the basal core lipid composition, both domains extensively reshape their membrane composition to overcome the inhospitable conditions. Here we discussed the efficiency of homeoviscous adaptations which may correlate with the different growth boundaries. At the observed boundaries it appears that bacterial core lipids are better accommodated to adjust the membrane fluidity, whereas archaeal core lipids are more efficient in rigidifying modifications. When looking into the membrane composition of the most extreme representatives of the domains, a consistent pattern appears at low pH and high temperature. Archaea form tetraether monolayer membranes that constitutes an adaptation mechanism of highly efficient rigidification and reduction of permeability. Interestingly, some of these typically archaeal features are also being implemented in bacterial hyperthermophiles and acidophiles. Examples are an increased level of ether-bonds, membrane spanning lipids, and a high level of methyl-branched BCFAs. It appears that both these features contribute to the fact that, e.g. both *Thermotogales* and *Aquificales* can survive higher growth temperatures than other bacterial hyperthermophiles. Typical membrane spanning lipids are also found in bacteria that thrive in acidic ecosystems. Here, however, these traits do not seem to improve the bacterial survival boundary at low pH. Despite this, it is conceivable that the cyclopentane rings do show correlation with the lowest pH survival range as formation of ring structures is also observed in bacterial lipids. In this case, it would support the hypothesized inferior survival boundaries to high temperature and low pH by implementing standard bacterial lipids (fatty acid-diester-lipids). On the other hand, at low temperatures it is observed that psychrophilic bacteria reduce their BCFA content as one of the main lipid modifications. This implies that at sub-freezing temperatures the effect of BCFAs to disturb the packing order near the interface of the bilayer is no longer sufficient to maintain fluidity. Because of that, facultative psychrophiles perhaps mainly implement unsaturations that cause a greater cross-sectional area of the lipids and less tight packing of the hydrocarbon chains than BCFAs. The isoprenoid hydrocarbon chains of archaeol which have an imperatively high number of methyl branches may thus be a disadvantageous characteristic at low temperatures. The methyl branches maintain a too dense packing which apparently cannot easily be compensated. In sharp contrast to bacteria, imitation of bacterial lipid features by archaea is thus far not observed in psychrophilic or alkaliphilic ecosystems. Despite this, both ester and ether lipid fatty acid phospholipids are detected in archaea, but there does not seem to be a correlation with either growth temperatures or pH from the analysed archaea thus far (Gattinger et al. 2002). Altogether, the deductions presented here are in line with the hypothesis that cytoplasmic membrane compositions are one of the main determining factors behind survival boundaries at extreme pH and temperatures.

## Electronic supplementary material

Below is the link to the electronic supplementary material.

Supplementary material 1 (DOCX 52 kb)

## Abbreviations

AA: Arachidonic acid
BCFA: Branched chain fatty acid
BMP: Bis-mono-acylglycero-phosphate
CL: Cardiolipin
DE: Diether
DHA: Docosahexaenoic acid
DGGGP: Di-*O*-geranylgeranylglycerylphosphate
EPA: Eicosapentaenoic acid
GDGT: Glyceroldialkyl-glycerol-tetraether
G-1-P: Glycerol-1-phosphate
G-3-P: Glycerol-3-phosphate
IPL: Intact polar lipid
MUFA: Monounsaturated fatty acid
PMF: Proton motive force
PUFA: Polyunsaturated fatty acid
SCFA: Short chain fatty acid
TE: Tetraether
UFA: Unsaturated fatty acid

## References

[CR1] Allen EE, Facciotti D, Bartlett DH (1999). Monounsaturated but not polyunsaturated fatty acids are required for growth of the deep-sea bacterium *Photobacterium profundum* SS9 at high pressure and low temperature. Appl Environ Microbiol.

[CR2] Auman AJ, Breezee JL, Gosink JJ, Kampfer P, Staley JT (2006). *Psychromonas ingrahamii* sp. nov., a novel gas vacuolate, psychrophilic bacterium isolated from Arctic polar sea ice. Int J Syst Evol Microbiol.

[CR4] Baker-Austin C, Dopson M (2007). Life in acid: pH homeostasis in acidophiles. Trends Microbiol.

[CR5] Balk M (2009). Isolation and characterization of a new CO-utilizing strain, *Thermoanaerobacter thermohydrosulfuricus* subsp. *carboxydovorans*, isolated from a geothermal spring in Turkey. Extremophiles.

[CR6] Bartlett DH (1992). Microbial life at high-pressures. Sci Prog.

[CR7] Blocher D, Gutermann R, Henkel B, Ring K (1990). Physicochemical characterization of tetraether lipids from *Thermoplasma acidophilum*. V. Evidence for the existence of a metastable state in lipids with acyclic hydrocarbon chains. Biochim Biophys Acta.

[CR8] Bowers KJ, Wiegel J (2011). Temperature and pH optima of extremely halophilic archaea: a mini-review. Extremophiles.

[CR9] Bowman JP, Cavanagh J, Austin JJ, Sanderson K (1996). Novel psychrobacter species from antarctic ornithogenic soils. Int J Syst Evol Microbiol.

[CR10] Boyd ES (2011). Temperature and pH controls on glycerol dibiphytanyl glycerol tetraether lipid composition in the hyperthermophilic crenarchaeon *Acidilobus sulfurireducens*. Extremophiles.

[CR11] Breezee J, Cady N, Staley JT (2004). Subfreezing growth of the sea ice bacterium “*Psychromonas ingrahamii*”. Microb Ecol.

[CR13] Brock TD, Freeze H (1969). *Thermus aquaticus* gen. n. and sp. n., a nonsporulating extreme thermophile. J Bacteriol.

[CR14] Campbell LL, Postgate JR (1965). Classification of the spore-forming sulfate-reducing bacteria. Bacteriol Rev.

[CR15] Cario A, Grossi V, Schaeffer P, Oger PM (2015). Membrane homeoviscous adaptation in the piezo-hyperthermophilic archaeon *Thermococcus barophilus*. Front Microbiol.

[CR16] Wirsen CO, Jannasch Holger W, Wakeham Stuart G, Canuel Elizabeth  A (1987). Membrane lipids of a psychrophilic and barophilic deep-sea bacterium. Curr Microbiol.

[CR17] Casadei MA, Manas P, Niven G, Needs E, Mackey BM (2002). Role of membrane fluidity in pressure resistance of *Escherichia coli* NCTC 8164. Appl Environ Microbiol.

[CR18] Castillo AM (2006). *Halorubrum orientale* sp. nov., a halophilic archaeon isolated from Lake Ejinor, Inner Mongolia, China. Int J Syst Evol Microbiol.

[CR19] Chan K, Leung O (1978). Nutrition and growth of the moderately halophilic bacteria *Micrococcus morrhuae* K-17 and *Micrococcus luteus* K-15. Microbios.

[CR20] Chang YY, Cronan JE (1999). Membrane cyclopropane fatty acid content is a major factor in acid resistance of *Escherichia coli*. Mol Microbiol.

[CR21] Chattopadhyay MK (2006). Mechanism of bacterial adaptation to low temperature. J Biosci.

[CR22] Chattopadhyay M, Jagannadham M, Vairamani M, Shivaji S (1997). Carotenoid pigments of an antarctic psychrotrophic bacterium *Micrococcus roseus*: temperature dependent biosynthesis, structure, and interaction with synthetic membranes. Biochem Biophys Res Commun.

[CR23] Chintalapati S, Kiran MD, Shivaji S (2004). Role of membrane lipid fatty acids in cold adaptation. Cell Mol Biol (Noisy-le-grand).

[CR183] Chugunov AO, Volynsky PE, Krylov NA, Boldyrev IA, Efremov RG (2014). Liquid but durable: molecular dynamics simulations explain the unique properties of archaeal-like membranes. Sci Rep.

[CR24] Clejan S, Krulwich TA, Mondrus KR, Seto-Young D (1986). Membrane lipid composition of obligately and facultatively alkalophilic strains of *Bacillus* spp. J Bacteriol.

[CR26] Dalmasso C, Oger P, Courtine D, Georges M, Takai K, Maignien L, Alain K (2016). Complete genome sequence of the hyperthermophilic and piezophilic archeon *Thermococcus piezophilus* CDGST, able to grow under extreme hydrostatic pressures. Genome Announcements.

[CR27] D’Amico S, Collins T, Marx J-C, Feller G, Gerday C (2006). Psychrophilic microorganisms: challenges for life. EMBO Rep.

[CR28] Damste JS, Rijpstra WI, Hopmans EC, Weijers JW, Foesel BU, Overmann J, Dedysh SN (2011). 13,16-Dimethyl octacosanedioic acid (iso-diabolic acid), a common membrane-spanning lipid of *Acidobacteria* subdivisions 1 and 3. Appl Environ Microbiol.

[CR29] Damsté JSS, Rijpstra WIC, Hopmans EC, Schouten S, Balk M, Stams AJM (2007). Structural characterization of diabolic acid-based tetraester, tetraether and mixed ether/ester, membrane-spanning lipids of bacteria from the order *Thermotogales*. Arch Microbiol.

[CR30] Dannenmuller O (2000). Membrane properties of archaeal macrocyclic diether phospholipids. Chemistry.

[CR31] Darland G, Brock TD (1971). *Bacillus acidocaldarius* sp. nov., an acidophilic thermophilic spore-forming bacterium. Microbiology.

[CR32] De Rosa M, Gambacorta A, Bu’lock JD (1974). Effects of pH and temperature on the fatty acid composition of *Bacillus acidocaldarius*. J Bacteriol.

[CR33] De Rosa M, Esposito E, Gambacorta A, Nicolaus B, Bu’Lock JD (1980). Effects of temperature on ether lipid composition of *Caldariella acidophila*. Phytochemistry.

[CR34] De Rosa M, Gambacorta A, Trincone A, Basso A, Zillig W, Holz I (1987). Lipids of *Thermococcus celer*, a sulfur-reducing archaebacterium: structure and biosynthesis. Syst Appl Microbiol.

[CR35] Dees SB, Carlone GM, Hollis D, Moss CW (1985). Chemical and phenotypic characteristics of *Flavobacterium thalpophilum* compared with those of other *Flavobacterium* and *Sphingobacterium* Species. Int J Syst Evol Microbiol.

[CR36] Denich TJ, Beaudette LA, Lee H, Trevors JT (2003). Effect of selected environmental and physico-chemical factors on bacterial cytoplasmic membranes. J Microbiol Methods.

[CR37] Drucker DB (1974). Chemotaxonomic fatty-acid fingerprints of some *Streptococci* with subsequent statistical analysis. Can J Microbiol.

[CR38] Elferink MG, de Wit JG, Driessen AJ, Konings WN (1994). Stability and proton-permeability of liposomes composed of archaeal tetraether lipids. Biochim Biophys Acta.

[CR39] Elsden SR, Hilton MG, Parsley KR, Self R (1980). The lipid fatty acids of proteolytic *Clostridia*. Microbiology.

[CR40] Escriba PV (2006). Membrane-lipid therapy: a new approach in molecular medicine. Trends Mol Med.

[CR41] Eze MO (1991). Phase transitions in phospholipid bilayers: lateral phase separations play vital roles in biomembranes. Biochem Educ.

[CR42] Feng J, Zhou P, Zhou Y-G, Liu S-J, Warren-Rhodes K (2005). *Halorubrum alkaliphilum* sp. nov., a novel haloalkaliphile isolated from a soda lake in Xinjiang, China. Int J Syst Evol Microbiol.

[CR43] Fozo EM, Quivey RG (2004). Shifts in the membrane fatty acid profile of *Streptococcus mutans* enhance survival in acidic environments. Appl Environ Microbiol.

[CR44] Franzmann PD, Liu Y, Balkwill DL, Aldrich HC, Conway de Macario E, Boone DR (1997). *Methanogenium frigidum* sp. nov., a psychrophilic, H2-using methanogen from Ace Lake, Antarctica. Int J Syst Bacteriol.

[CR46] Freier D, Mothershed CP, Wiegel J (1988). Characterization of *Clostridium thermocellum* JW20. Appl Environ Microbiol.

[CR47] Gabriel JL, Chong PL (2000). Molecular modeling of archaebacterial bipolar tetraether lipid membranes. Chem Phys Lipids.

[CR48] Gattinger A, Schloter M, Munch JC (2002). Phospholipid etherlipid and phospholipid fatty acid fingerprints in selected euryarchaeotal monocultures for taxonomic profiling. FEMS Microbiol Lett.

[CR49] Gibson JA, Miller MR, Davies NW, Neill GP, Nichols DS, Volkman JK (2005). Unsaturated diether lipids in the psychrotrophic archaeon *Halorubrum lacusprofundi*. Syst Appl Microbiol.

[CR50] Gilmore SF, Yao AI, Tietel Z, Kind T, Facciotti MT, Parikh AN (2013). Role of squalene in the organization of monolayers derived from lipid extracts of *Halobacterium salinarum*. Langmuir.

[CR51] Giotis ES, McDowell DA, Blair IS, Wilkinson BJ (2007). Role of branched-chain fatty acids in pH stress tolerance in *Listeria monocytogenes*. Appl Environ Microbiol.

[CR52] Girard AE (1971). A comparative study of the fatty acids of some *Micrococci*. Can J Microbiol.

[CR53] Gliozzi A, Paoli G, De Rosa M, Gambacorta A (1983). Effect of isoprenoid cyclization on the transition temperature of lipids in thermophilic archaebacteria. Biochimica et Biophysica Acta (BBA)-Biomembr.

[CR54] Grant WD, Pinch G, Harris JE, De Rosa M, Gambacorta A (1985). polar lipids in methanogen taxonomy. Microbiology.

[CR55] Gruner SM, Cullis PR, Hope MJ, Tilcock CP (1985). Lipid polymorphism: the molecular basis of nonbilayer phases. Ann Rev Biophys Biophys Chem.

[CR56] Gruszecki WI, Strzałka K (2005). Carotenoids as modulators of lipid membrane physical properties. Biochimica et Biophysica Acta (BBA)-Mol Basis Dis.

[CR57] Guan Z, Tian B, Perfumo A, Goldfine H (2013). The polar lipids of *Clostridium psychrophilum*, an anaerobic psychrophile. Biochim Biophys Acta.

[CR58] Hafenbradl D, Keller M, Thiericke R, Stetter KO (1993). A novel unsaturated archaeal ether core lipid from the hyperthermophile *Methanopyrus kandleri*. Syst Appl Microbiol.

[CR59] Hafenbradl D, Keller M, Stetter KO (1996). Lipid analysis of *Methanopyrus kandleri*. FEMS Microbiol Lett.

[CR60] Haines TH (2001). Do sterols reduce proton and sodium leaks through lipid bilayers?. Prog Lipid Res.

[CR61] Hauß T, Dante S, Dencher NA, Haines TH (2002). Squalane is in the midplane of the lipid bilayer: implications for its function as a proton permeability barrier. Biochimica et Biophysica Acta (BBA)-Bioenerg.

[CR62] Heinen W, Klein HP, Volkmann CM (1970). Fatty acid composition of *Thermus aquaticus* at different growth temperatures. Arch Mikrobiol.

[CR63] Henssen A, Schnepf E (1967). Studies on thermophilic *Actinomycetes*. Archiv für Mikrobiologie.

[CR64] Herrero AA, Gomez RF, Roberts MF (1982). Ethanol-induced changes in the membrane lipid composition of *Clostridium thermocellum*. Biochimica et Biophysica Acta (BBA)-Biomembranes.

[CR65] Holmes B, Hollis DG, Steigerwalt AG, Pickett MJ, Brenner DJ (1983). *Flavobacterium thalpophilum*, a new species recovered from human clinical material. Int J Syst Evol Microbiol.

[CR66] Hu L (2008). *Halorubrum luteum* sp. nov., isolated from Lake Chagannor, Inner Mongolia, China. Int J Syst Evol Microbiol.

[CR67] Huber R, Woese CR, Langworthy TA, Fricke H, Stetter KO (1989). *Thermosipho africanus* gen. nov., represents a new genus of thermophilic eubacteria within the “*Thermotogales*”. Syst Appl Microbiol.

[CR68] Huber R (1992). *Aquifex pyrophilus* gen. nov. sp. nov., represents a novel group of marine hyperthermophilic hydrogen-oxidizing bacteria. Syst Appl Microbiol.

[CR69] Huston AL, Methe B, Deming JW (2004). Purification, characterization, and sequencing of an extracellular cold-active aminopeptidase produced by marine psychrophile *Colwellia psychrerythraea* strain 34H. Appl Environ Microbiol.

[CR70] Jackson TJ, Ramaley RF, Meinschein WG (1972). Fatty acids of a non-pigmented, thermophilic bacterium similar to *Thermus aquaticus*. Archiv für Mikrobiologie.

[CR71] Jackson S, Calos M, Myers A, Self WT (2006). Analysis of proline reduction in the nosocomial pathogen *Clostridium difficile*. J Bacteriol.

[CR72] Jagannadham MV, Chattopadhyay MK, Subbalakshmi C, Vairamani M, Narayanan K, Rao CM, Shivaji S (2000). Carotenoids of an Antarctic psychrotolerant bacterium, *Sphingobacterium antarcticus*, and a mesophilic bacterium, *Sphingobacterium multivorum*. Arch Microbiol.

[CR74] Jahnke LL (2001). Signature lipids and stable carbon isotope analyses of Octopus Spring hyperthermophilic communities compared with those of *Aquificales* representatives. Appl Environ Microbiol.

[CR75] Janto B (2011). Genome of alkaliphilic *Bacillus pseudofirmus* OF4 reveals adaptations that support the ability to grow in an external pH range from 7.5 to 11.4. Environ Microbiol.

[CR76] Jantzen E, Bergan T, Bøvre K (1974). Gas chromatography of bacterial whole cell methanolysates. Acta Pathologica Microbiologica Scandinavica Section B Microbiol Immunol.

[CR77] Jebbar M, Franzetti B, Girard E, Oger P (2015). Microbial diversity and adaptation to high hydrostatic pressure in deep-sea hydrothermal vents prokaryotes. Extremophiles.

[CR78] Jung S, Zeikus JG, Hollingsworth RI (1994). A new family of very long chain alpha, omega-dicarboxylic acids is a major structural fatty acyl component of the membrane lipids of Thermoanaerobacter ethanolicus 39E. J Lipid Res.

[CR79] Kaneda T (1968). Fatty acids in the genus *Bacillus.* II. Similarity in the fatty acid compositions of *Bacillus thuringiensis*, *Bacillus anthracis*, and *Bacillus cereus*. J Bacteriol.

[CR80] Kaneda T (1977). Fatty acids of the genus *Bacillus*: an example of branched-chain preference. Bacteriol Rev.

[CR81] Kaneda T (1991). Iso- and anteiso-fatty acids in bacteria: biosynthesis, function, and taxonomic significance. Microbiol Rev.

[CR82] Kaneda T, Smith EJ, Naik DN (1983). Fatty acid composition and primer specificity of de novo fatty acid synthetase in *Bacillus globispores*, *Bacillus insolitus*, and *Bacillus psychrophilus*. Can J Microbiol.

[CR83] Kaneshiro SM, Clark DS (1995). Pressure effects on the composition and thermal behavior of lipids from the deep-sea thermophile *Methanococcus jannaschii*. J Bacteriol.

[CR84] Kashefi K, Holmes DE, Reysenbach A-L, Lovley DR (2002). Use of Fe(III) as an electron acceptor to recover previously uncultured hyperthermophiles: isolation and characterization of *Geothermobacterium ferrireducens* gen. nov., sp. nov. Appl Environ Microbiol.

[CR85] K-i Suzuki, Collins MD, Iijima E, Komagata K (1988). Chemotaxonomic characterization of a radiotolerant bacterium, *Arthrobacter radiotolerans*: description of *Rubrobacter radiotolerans* gen. nov., comb. nov. FEMS Microbiol Lett.

[CR86] Kim BH, Kim S, Kim HG, Lee J, Lee IS, Park YK (2005). The formation of cyclopropane fatty acids in *Salmonella enterica serovar Typhimurium*. Microbiology.

[CR87] Kiran MD (2004). Psychrophilic *Pseudomonas syringae* requires trans-monounsaturated fatty acid for growth at higher temperature. Extremophiles.

[CR88] Kloos WE, Schleifer KH (1975). Isolation and Characterization of Staphylococci from Human Skin II. Descriptions of four new species: *Staphylococcus warneri, Staphylococcus capitis*, *Staphylococcus hominis,* and S*taphylococcus simulans*. Int J Syst Evol Microbiol.

[CR89] Knoblauch C, Sahm K, Jorgensen BB (1999). Psychrophilic sulfate-reducing bacteria isolated from permanently cold arctic marine sediments: description of *Desulfofrigus oceanense* gen. nov., sp. nov., *Desulfofrigus fragile* sp. nov., *Desulfofaba gelida* gen. nov., sp. nov., *Desulfotalea psychrophila* gen. nov., sp. nov. and *Desulfotalea arctica* sp. nov. Int J Syst Bacteriol.

[CR90] Koehler TM (2009). *Bacillus anthracis* physiology and genetics. Mol Aspects Med.

[CR91] Koga Y (2012). Thermal adaptation of the archaeal and bacterial lipid membranes. Archaea.

[CR92] Koga Y, Morii H (2005). Recent advances in structural research on ether lipids from archaea including comparative and physiological aspects. Biosci Biotechnol Biochem.

[CR93] Konings WN, Albers SV, Koning S, Driessen AJ (2002). The cell membrane plays a crucial role in survival of bacteria and archaea in extreme environments. Antonie Van Leeuwenhoek.

[CR94] Krulwich TA (2006). Alkaliphilic Prokaryotes. Prokaryotes.

[CR95] Kurr M (1991). *Methanopyrus kandleri*, gen. and sp. nov. represents a novel group of hyperthermophilic methanogens, growing at 110 C. Arch Microbiol.

[CR96] Lai D, Springstead JR, Monbouquette HG (2008). Effect of growth temperature on ether lipid biochemistry in *Archaeoglobus fulgidus*. Extremophiles.

[CR97] Langworthy TA, Holzer G, Zeikus JG, Tornabene TG (1983). Iso- and anteiso-branched glycerol diethers of the thermophilic anaerobe *Thermodesulfotobacterium commune*. Syst Appl Microbiol.

[CR99] Lanzotti V, Nicolaus B, Trincone A, De Rosa M, Grant WD, Gambacorta A (1989). A complex lipid with a cyclic phosphate from the archaebacterium *Natronococcus occultus*. Biochimica et Biophysica Acta (BBA)-Lipids Lipid Metabol.

[CR101] Lee S, Kang S, Kim N, Jung SH (2002). Structural analyses of the novel phosphoglycolipids containing the unusual very long bifunctional acyl chain, alpha, omega-13,16-dimethyloctacosanedioate in Thermoanaerobacter ethanolicus. Bull Korean Chem Soc.

[CR102] Li Y, Engle M, Weiss N, Mandelco L, Wiegel J (1994). *Clostridium thermoalcaliphilum* sp. nov., an anaerobic and thermotolerant facultative alkaliphile. Int J Syst Bacteriol.

[CR103] Loginova LG, Egorova LA, Golovacheva RS, Seregina LM (1984). *Thermus ruber* sp. nov., nom. rev. Int J Syst Evol Microbiol.

[CR104] Macalady JL, Vestling MM, Baumler D, Boekelheide N, Kaspar CW, Banfield JF (2004). Tetraether-linked membrane monolayers in Ferroplasma spp: a key to survival in acid. Extremophiles.

[CR105] Mangelsdorf K, Zink K-G, Birrien J-L, Toffin L (2005). A quantitative assessment of pressure dependent adaptive changes in the membrane lipids of a piezosensitive deep sub-seafloor bacterium. Org Geochem.

[CR106] Marr AG, Ingraham JL (1962). Effect of temperature on the composition of fatty acids in *Escherichia Coli*. J Bacteriol.

[CR107] Mathai JC, Sprott GD, Zeidel ML (2001). Molecular mechanisms of water and solute transport across archaebacterial lipid membranes. J Biol Chem.

[CR108] Matsubara H, Goto K, Matsumura T, Mochida K, Iwaki M, Niwa M, Yamasato K (2002). *Alicyclobacillus acidiphilus* sp. nov., a novel thermo-acidophilic, omega-alicyclic fatty acid-containing bacterium isolated from acidic beverages. Int J Syst Evol Microbiol.

[CR109] Matsuno Y (2009). Effect of growth temperature and growth phase on the lipid composition of the archaeal membrane from *Thermococcus kodakaraensis*. Biosci Biotechnol Biochem.

[CR110] McElhaney RN, Souza KA (1976). The relationship between environmental temperature, cell growth and the fluidity and physical state of the membrane lipids in *Bacillus stearothermophilus*. Biochimica et Biophysica Acta (BBA)-Nucleic Acids Protein Synth.

[CR111] Morii H, Yagi H, Akutsu H, Nomura N, Sako Y, Koga Y (1999). A novel phosphoglycolipid archaetidyl(glucosyl)inositol with two sesterterpanyl chains from the aerobic hyperthermophilic archaeon Aeropyrum pernix K1. Biochimica et Biophysica Acta (BBA)-Mol Cell Biol Lipids.

[CR112] Moss CW, Dowell VR, Farshtchi D, Raines LJ, Cherry WB (1969). Cultural characteristics and fatty acid composition of propionibacteria. J Bacteriol.

[CR113] Moule AL, Wilkinson SG (1987). Polar lipids, fatty acids, and isoprenoid quinones of *Alteromonas putrefaciens* (*Shewanella putrefaciens*). Syst Appl Microbiol.

[CR114] Mykytczuk NC, Trevors JT, Leduc LG, Ferroni GD (2007). Fluorescence polarization in studies of bacterial cytoplasmic membrane fluidity under environmental stress. Prog Biophys Mol Biol.

[CR115] Mykytczuk NC, Trevors JT, Ferroni GD, Leduc LG (2010). Cytoplasmic membrane fluidity and fatty acid composition of *Acidithiobacillus ferrooxidans* in response to pH stress. Extremophiles.

[CR116] Nakamura L (1984). *Bacillus psychrophilus* sp. nov., nom. rev. Int J Syst Evol Microbiol.

[CR117] Namwong S, Tanasupawat S, Visessanguan W, Kudo T, Itoh T (2007). *Halococcus thailandensis* sp. nov., from fish sauce in Thailand. Int J Syst Evol Microbiol.

[CR118] Nichols DS, Miller MR, Davies NW, Goodchild A, Raftery M, Cavicchioli R (2004). Cold adaptation in the Antarctic Archaeon *Methanococcoides burtonii* involves membrane lipid unsaturation. J Bacteriol.

[CR119] Nogi Y, Kato C (1999). Taxonomic studies of extremely barophilic bacteria isolated from the Mariana Trench and description of *Moritella yayanosii* sp. nov., a new barophilic bacterial isolate. Extremophiles.

[CR120] Nonomura H, Ohara Y (1960). Distribution of the actinomycetes in soil. IV. The isolation and classification of the genus Streptosporangium. J Ferment Technol.

[CR121] O’Donnel AG, Nahaie MR, Goodfellow M, Minnikin DE, Hájek V (1985). Numerical analysis of fatty acid profiles in the identification of Staphylococci. Microbiology.

[CR122] Oger PM, Cario A (2013). Adaptation of the membrane in Archaea. Biophys Chem.

[CR123] Oger PM, Jebbar M (2010). The many ways of coping with pressure. Res Microbiol.

[CR124] Oshima T, Imahori K (1974). Description of *Thermus thermophilus* (Yoshida and Oshima) comb. nov., a nonsporulating thermophilic bacterium from a Japanese thermal spa. Int J Syst Evol Microbiol.

[CR125] Oshima M, Miyagawa A (1974). Comparative studies on the fatty acid composition of moderately and extremely thermophilic bacteria. Lipids.

[CR127] Patel BKC, Skerratt JH, Nichols PD (1991). The phospholipid ester-linked fatty acid composition of thermophilic bacteria. Syst Appl Microbiol.

[CR128] Pivnick H (1955). *Pseudomonas rubescens*, a new species from soluble oil emulsions. J Bacteriol.

[CR129] Preston CM, Wu KY, Molinski TF, DeLong EF (1996). A psychrophilic crenarchaeon inhabits a marine sponge: *Cenarchaeum symbiosum* gen. nov., sp. nov. Proc Natl Acad Sci.

[CR130] Prowe SG, Antranikian G (2001). *Anaerobranca gottschalkii* sp. nov., a novel thermoalkaliphilic bacterium that grows anaerobically at high pH and temperature. Int J Syst Evol Microbiol.

[CR131] Rainey FA, Ward-Rainey N, Kroppenstedt RM, Stackebrandt E (1996). The genus *Nocardiopsis* represents a phylogenetically coherent taxon and a distinct actinomycete lineage: proposal of *Nocardiopsaceae* fam. nov. Int J Syst Bacteriol.

[CR132] Ray PH, White DC, Brock TD (1971). Effect of growth temperature on the lipid composition of *Thermus aquaticus*. J Bacteriol.

[CR133] Roger P, Delettre J, Bouix M, Beal C (2011). Characterization of *Streptococcus salivarius* growth and maintenance in artificial saliva. J Appl Microbiol.

[CR134] Russell NJ (1990). Cold adaptation of microorganisms. Philos Trans R Soc Lond B Biol Sci.

[CR135] Russell NJ (1997). Psychrophilic bacteria–molecular adaptations of membrane lipids. Comp Biochem Physiol A Physiol.

[CR136] Russell NJ, Fukunaga N (1990). A comparison of thermal adaptation of membrane lipids in psychrophilic and thermophilic bacteria. FEMS Microbiol Lett.

[CR137] Sako Y (1996). *Aeropyrum pernix* gen. nov., sp. nov., a novel aerobic hyperthermophilic archaeon growing at temperatures up to 100 degrees C. Int J Syst Bacteriol.

[CR138] Sanghoo Lee SK, Kim Jai Neung, Jung Seunho (2002). Structural analyses of the novel phosphoglycolipids containing the unusual very long bifunctional acyl chain, α, ω-13-16-dimethyloctacosanedioate in Thermoanaerobacter ethanolicus. Bull Korean Chem Soc.

[CR139] Schleifer KH, Kilpper-Bälz R (1984). Transfer of *Streptococcus faecalis* and *Streptococcus faecium* to the Genus *Enterococcus* nom. rev. as *Enterococcus faecalis* comb. nov. and *Enterococcus faecium* comb. nov. Int J Syst Evol Microbiol.

[CR140] Schleper C (1995). *Picrophilus* gen. nov., fam. nov.: a novel aerobic, heterotrophic, thermoacidophilic genus and family comprising archaea capable of growth around pH 0. J Bacteriol.

[CR141] Shimada H, Nemoto N, Shida Y, Oshima T, Yamagishi A (2008). Effects of pH and temperature on the composition of polar lipids in *Thermoplasma acidophilum* HO-62. J Bacteriol.

[CR142] Shivaji S, Prakash JS (2010). How do bacteria sense and respond to low temperature?. Arch Microbiol.

[CR143] Shivaji S, Rao NS, Saisree L, Sheth V, Reddy GSN, Bhargava PM (1988). Isolation and identification of *Micrococcus roseus* and *Planococcus* sp. from schirmacher oasis, antarctica. J Biosci.

[CR144] Shivaji S (1992). *Sphingobacterium antarcticus* sp. nov., a psychrotrophic bacterium from the soils of Schirmacher Oasis, Antarctica. Int J Syst Evol Microbiol.

[CR145] Shtarkman YM, Kocer ZA, Edgar R, Veerapaneni RS, D’Elia T, Morris PF, Rogers SO (2013). Subglacial Lake Vostok (Antarctica) accretion ice contains a diverse set of sequences from aquatic, marine and sediment-inhabiting bacteria and eukarya. PLoS One.

[CR146] Sinensky M (1971). Temperature control of phospholipid biosynthesis in *Escherichia coli*. J Bacteriol.

[CR147] Sinensky M (1974). Homeoviscous adaptation-a homeostatic process that regulates the viscosity of membrane lipids in *Escherichia coli*. Proc Natl Acad Sci USA.

[CR148] Spring S, Merkhoffer B, Weiss N, Kroppenstedt RM, Hippe H, Stackebrandt E (2003). Characterization of novel psychrophilic clostridia from an Antarctic microbial mat: description of *Clostridium frigoris* sp. nov., *Clostridium lacusfryxellense* sp. nov., *Clostridium bowmanii* sp. nov. and *Clostridium psychrophilum* sp. nov. and reclassification of *Clostridium laramiense* as *Clostridium estertheticum subsp. laramiense* subsp. nov. Int J Syst Evol Microbiol.

[CR149] Sprott GD, Meloche M, Richards JC (1991). Proportions of diether, macrocyclic diether, and tetraether lipids in *Methanococcus jannaschii* grown at different temperatures. J Bacteriol.

[CR150] Sprott GD, Agnew BJ, Patel GB (1997). Structural features of ether lipids in the archaeobacterial thermophiles *Pyrococcus furiosus, Methanopyrus kandleri, Methanothermus fervidus*, and *Sulfolobus acidocaldarius*. Can J Microbiol.

[CR151] Su Y, Rhee MS, Ingram LO, Shanmugam K (2011). Physiological and fermentation properties of *Bacillus coagulans* and a mutant lacking fermentative lactate dehydrogenase activity. J Ind Microbiol Biotechnol.

[CR152] Sugai A, Uda I, Itoh YH, Itoh T (2004). The core lipid composition of the 17 strains of hyperthermophilic archaea, *Thermococcales*. J Oleo Sci.

[CR153] Suutari M, Laakso S (1994). Microbial fatty acids and thermal adaptation. Crit Rev Microbiol.

[CR154] Takai K, Sugai A, Itoh T, Horikoshi K (2000). *Palaeococcus ferrophilus* gen. nov., sp. nov., a barophilic, hyperthermophilic archaeon from a deep-sea hydrothermal vent chimney. Int J Syst Evol Microbiol.

[CR155] Takai K (2008). Cell proliferation at 122 C and isotopically heavy CH4 production by a hyperthermophilic methanogen under high-pressure cultivation. Proc Natl Acad Sci.

[CR156] Taylor J, Parkes RJ (1983). The cellular fatty acids of the sulphate-reducing bacteria, *Desulfobacter* sp., Desulfobulbus sp. and Desulfovibrio desulfuricans. Microbiology.

[CR157] Thies E, Jenkins T, Stutzenberger F (1994). Effects of the detergent Tween 80 on *Thermomonospora curvata*. World J Microbiol Biotechnol.

[CR158] Tindall BJ, Ross HNM, Grant WD (1984). *Natronobacterium* gen. nov. and *Natronococcus* gen. nov., Two New Genera of Haloalkaliphilic Archaebacteria. Syst Appl Microbiol.

[CR159] Tsu I-H, Huang C-Y, Garcia J-L, Patel CBK, Cayol J-L, Baresi L, Mah AR (1998). Isolation and characterization of *Desulfovibrio senezii* sp. nov., a halotolerant sulfate reducer from a solar saltern and phylogenetic confirmation of *Desulfovibrio fructosovorans* as a new species. Arch Microbiol.

[CR160] Uda I, Sugai A, Itoh YH, Itoh T (2001). Variation in molecular species of polar lipids from *Thermoplasma acidophilum* depends on growth temperature. Lipids.

[CR161] Uda I, Sugai A, Itoh YH, Itoh T (2004). Variation in molecular species of core lipids from the order *Thermoplasmales* strains depends on the growth temperature. J Oleo Sci.

[CR162] Ueki A, Suto T (1979). Cellular fatty acid composition of sulfate-reducing bacteria. J General Appl Microbiol.

[CR163] Ulrih NP, Gmajner D, Raspor P (2009). Structural and physicochemical properties of polar lipids from thermophilic archaea. Appl Microbiol Biotechnol.

[CR164] van de Vossenberg JL, Driessen AJ, Zillig W, Konings WN (1998). Bioenergetics and cytoplasmic membrane stability of the extremely acidophilic, thermophilic archaeon Picrophilus oshimae. Extremophiles.

[CR165] van de Vossenberg JL, Driessen AJ, Konings NW (1998). The essence of being extremophilic: the role of the unique archaeal membrane lipids. Extremophiles.

[CR166] Wakao N (1994). *Acidiphilium multivorum* sp. nov., an acidophilic chemoorganotrophic bacterium from pyritic acid mine drainage. J General Appl Microbiol.

[CR167] Wan X, Peng Y-F, Zhou X-R, Gong Y-M, Huang F-H, Moncalián G (2016). Effect of cerulenin on fatty acid composition and gene expression pattern of DHA-producing strain *Colwellia psychrerythraea* strain 34H. Microb Cell Fact.

[CR169] Weijers JW (2006). Membrane lipids of mesophilic anaerobic bacteria thriving in peats have typical archaeal traits. Environ Microbiol.

[CR170] Wichlacz PL, Unz RF, Langworthy TA (1986). *Acidiphilium angustum* sp. nov., *Acidiphilium facilis* sp. nov., and *Acidiphilium rubrum* sp. nov.: acidophilic heterotrophic bacteria isolated from acidic coal mine drainage. Int J Syst Evol Microbiol.

[CR171] Xu Y, Zhou P, Tian X (1999). Characterization of two novel haloalkaliphilic archaea *Natronorubrum bangense* gen. nov., sp. nov. and *Natronorubrum tibetense* gen. nov., sp. nov. Int J Syst Evol Microbiol.

[CR172] Xu Y, Wang Z, Xue Y, Zhou P, Ma Y, Ventosa A, Grant WD (2001). *Natrialba hulunbeirensis* sp. nov. and *Natrialba chahannaoensis* sp. nov., novel haloalkaliphilic archaea from soda lakes in Inner Mongolia Autonomous Region, China. Int J Syst Evol Microbiol.

[CR173] Yamauchi K, Doi K, Yoshida Y, Kinoshita M (1993). Archaebacterial lipids: highly proton-impermeable membranes from 1,2-diphytanyl-sn-glycero-3-phosphocholine. Biochim Biophys Acta.

[CR174] Yano Y, Nakayama A, Ishihara K, Saito H (1998). Adaptive changes in membrane lipids of barophilic bacteria in response to changes in growth pressure. Appl Environ Microbiol.

[CR175] Yayanos AA (1986). Evolutional and ecological implications of the properties of deep-sea barophilic bacteria. Proc Natl Acad Sci.

[CR176] Yayanos AA, Dietz AS, Van Boxtel R (1981). Obligately barophilic bacterium from the Mariana Trench. Proc Natl Acad Sci.

[CR177] Ye Q, Roh Y, Carroll SL, Blair B, Zhou J, Zhang CL, Fields MW (2004). Alkaline anaerobic respiration: isolation and characterization of a novel alkaliphilic and metal-reducing bacterium. Appl Environ Microbiol.

[CR178] Yokoyama A, Shizuri Y, Hoshino T, Sandmann G (1996). Thermocryptoxanthins: novel intermediates in the carotenoid biosynthetic pathway of *Thermus thermophilus*. Arch Microbiol.

[CR179] Yoshinaka T, Yano K, Yamaguchi H (1973). Isolation of highly radioresistant bacterium, *Arthrobacter radiotolerans* nov. sp. Agric Biol Chem.

[CR180] Yuk HG, Marshall DL (2004). Adaptation of *Escherichia coli* O157:H7 to pH alters membrane lipid composition, verotoxin secretion, and resistance to simulated gastric fluid acid. Appl Environ Microbiol.

[CR181] Zeng X (2009). *Pyrococcus* CH1, an obligate piezophilic hyperthermophile: extending the upper pressure-temperature limits for life. ISME J.

[CR182] Zsiros O, Varkonyi Z, Kovacs A, Farkas T, Gombos Z, Garab G (2000). Induction of polyunsaturated fatty-acid synthesis enhances tolerance of a cyanobacterium, *Cylindrospermopsis raciborskii,* to low-temperature photoinhibition. Indian J Biochem Biophys.

