# Supplementary material for: Adaptations of archaeal and bacterial membranes to variations in temperature, pH and pressure
Source: Extremophiles. 2017 May 15;21(4):651–70. doi: 10.1007/s00792-017-0939-x (PMC5487899; doi:10.1007/s00792-017-0939-x)
Supplement: Supplementary file 1 — Supplementary material 1 (DOCX 52 kb) [file 792_2017_939_MOESM1_ESM.docx]

**Adaptations of Archaeal and Bacterial Membranes to Variations in Temperature, pH and Pressure**

**Extremophiles**

Melvin F. Siliakus^1^, John van der Oost^1^, Servé W. M. Kengen^1^

^1^ Laboratory of Microbiology, Wageningen University and Research Centre, Stippeneng 4, 6708 WE, Wageningen, the Netherlands

Corresponding author: [melvin.siliakus@wur.nl](mailto:melvin.siliakus@wur.nl)

Electronic supplementary material: ESM_1

**Table S1 Bacterial temperature optima versus branched chain fatty acid compositions**

| **#** | **BCFA %** | **Topt (^o^C)** | **Species** | **BCFA ref** | **Temp ref** |
| --- | --- | --- | --- | --- | --- |
| 1 | 0 | 15 | *Psychrobacter frigidicola* | (Bowman et al. 1996) | (Bowman et al. 1996) |
| 2 | 0 | 10 | *Desulfotalea psychrophila* | (Knoblauch et al. 1999) | (Knoblauch et al. 1999) |
| 3 | 1 | 10 | *Colwellia psychrerythraea* | (Wan et al. 2016) | (Wan et al. 2016) |
| 4 | 4,5 | 5 | *Psychromonas ingrahamii* | (Auman et al. 2006) | (Breezee et al. 2004) |
| 5 | 11 | 30 | *Micrococcus roseus* | (Girard 1971) | (Shivaji et al.) |
| 6 | 17 | 25 | *Sphingobacterium antarcticus* | (Jagannadham et al. 2000) | (Shivaji et al. 1992) |
| 7 | 19 | 37 | *Streptococcus faecalis* | (Drucker 1974) | (Schleifer and Kilpper-Bälz 1984) |
| 8 | 26 | 37 | *Clostridium difficile* | (Elsden et al. 1980) | (Jackson et al. 2006) |
| 9 | 28 | 27 | *Pseudomonas rubescens* | (Moule and Wilkinson 1987) | (Pivnick 1955) |
| 10 | 31 | 37 | *Flavobacterium thalpophilum* | (Dees et al. 1985) | (Holmes et al. 1983) |
| 11 | 32 | 28 | *Streptosporangium album* | (Kaneda 1991) | (Nonomura and Ohara 1960) |
| 12 | 32 | 35 | *Streptosporangium vulgare* | (Kaneda 1991) | (Nonomura and Ohara 1960) |
| 13 | 33 | 55 | *Desulfotomaculum nigrificans* | (Ueki and Suto 1979) | (Campbell and Postgate 1965) |
| 14 | 38 | 37 | *Streptococcus salivarius* | (Drucker 1974) | (Roger et al. 2011) |
| 15 | 40 | 65 | *Bacillus stearothermophilus* | (McElhaney and Souza 1976) | (McElhaney and Souza 1976) |
| 16 | 41 | 37 | *Streptococcus agalactiae* | (Drucker 1974) | ATCC |
| 17 | 45 | 37 | *Clostridium sordellii* | (Elsden et al. 1980) | Dsmz |
| 18 | 49 | 37 | *Staphylococcus capitis* | (O'Donnel et al. 1985) | (Kloos and Schleifer 1975) |
| 19 | 51 | 27 | *Actinoplanes philippinensis* | (Kaneda 1991) | ATCC |
| 20 | 52 | 34 | *Desulfovibrio africanus* | (Ueki and Suto 1979) | (Tsu et al. 1998) |
| 21 | 53 | 37 | *Desulfovibrio vulgaris* | (Ueki and Suto 1979) | ATCC |
| 22 | 55 | 37 | *Bacteroides vulgatus* | (Kaneda 1991) | Dsmz |
| 23 | 55 | 37 | *Streptococcus pyogenes* | (Drucker 1974) | Dsmz |
| 24 | 57 | 37 | *Propionibacterium acnes* | (Moss et al. 1969) | ATCC |
| 25 | 59 | 62.5 | *Bacillus acidocaldarius* | (De Rosa et al. 1974) | (Darland and Brock 1971) |
| 26 | 62 | 60 | *Thermus ruber* | (Loginova et al. 1984) | (Loginova et al. 1984) |
| 27 | 72 | 62 | *Thermomonospora curvata* | (Kaneda 1991) | (Henssen and Schnepf 1967; Thies et al. 1994) |
| 28 | 73 | 30 | *Bacillus pumilus* | (Kaneda 1977) | Dsmz |
| 29 | 73 | 60 | *Clostridium thermocellum* | (Herrero et al. 1982) | (Freier et al. 1988) |
| 30 | 75 | 30 | *Desulfovibrio desulfuricans* | (Taylor and Parkes 1983) | (Taylor and Parkes 1983) |
| 31 | 75 | 32 | *Staphylococcus simulans* | (O'Donnel et al. 1985) | (Kloos and Schleifer 1975) |
| 32 | 75 | 34 | *Staphylococcus hominis* | (O'Donnel et al. 1985) | (Kloos and Schleifer 1975) |
| 33 | 79 | 30 | *Bacillus cereus* | (Kaneda 1968) | ATCC |
| 34 | 80 | 37 | *Bacillus anthracis* | (Kaneda 1968) | (Koehler 2009) |
| 35 | 81 | 37 | *Staphylococcus aureus* | (O'Donnel et al. 1985) | Dsmz |
| 36 | 81 | 32 | *Staphylococcus epidermidis* | (O'Donnel et al. 1985) | Dsmz |
| 37 | 82 | 47 | *Arthrobacter radiotolerans* | (Suzuki et al. 1988) | (Yoshinaka et al. 1973) |
| 38 | 84 | 35 | *Staphylococcus haemolyticus* | (O'Donnel et al. 1985) | Bergey’s |
| 39 | 84 | 70 | *Thermodesulfotobacterium commune* | (Langworthy et al. 1983) | (Langworthy et al. 1983) |
| 40 | 87 | 30 | *Micrococcus luteus* | (Jantzen et al. 1974) | (Chan and Leung 1978) |
| 41 | 89 | 52.5 | *Bacillus coagulans* | (Kaneda 1991) | (Su et al. 2011) |
| 42 | 89 | 30 | *Bacillus megaterium* | (Kaneda 1991) | Bergey’s |
| 43 | 91 | 71 | *Thermus aquaticus* | (Jackson et al. 1972) | (Brock and Freeze 1969) |
| 44 | 92 | 25 | *Bacillus psychrophilus* | (Kaneda et al. 1983) | (Nakamura 1984) |
| 45 | 95 | 33,5 | *Bacillus subtilis* | (Kaneda 1977) | Dsmz |
| 46 | 98 | 70 | *Thermoanaerobacter thermohydrosulfuricus subsp. carboxydovorans* | (Balk et al. 2009) | (Balk et al. 2009) |
| 47 | 99 | 72 | *Thermus thermophilus* | (Oshima and Miyagawa 1974) | (Oshima and Imahori 1974) |
| 48 | 99 | 28 | *Micrococcus conglomeratus* | (Jantzen et al. 1974) | Dsmz |

**Abbreviations**: ATCC (American type culture collection) bacteriology collection, DSMZ; Deutsche Sammlung von Microorganismen und Zellkulturen, Bergey’s; Bergey’s manual of systematic bacteriology
